# Data-Dependent Acquisition and Database-Driven Efficient Peak Annotation for the Comprehensive Profiling and Characterization of the Multicomponents from Compound Xueshuantong Capsule by UHPLC/IM-QTOF-MS

**DOI:** 10.3390/molecules24193431

**Published:** 2019-09-21

**Authors:** Tiantian Zuo, Yuexin Qian, Chunxia Zhang, Yuxi Wei, Xiaoyan Wang, Hongda Wang, Ying Hu, Weiwei Li, Xiaohui Wu, Wenzhi Yang

**Affiliations:** 1 Tianjin State Key Laboratory of Modern Chinese Medicine, Tianjin University of Traditional Chinese Medicine, 312 Anshanxi Road, Tianjin 300193, China; 13553170361@163.com (T.Z.); qyx15232238286@163.com (Y.Q.); 18202669277@163.com (C.Z.); 14741372177@163.com (X.W.); 17862987156@163.com (H.W.); huying3916@163.com (Y.H.); lww11413@163.com (W.L.); 2 Tianjin Key Laboratory of TCM Chemistry and Analysis, Tianjin University of Traditional Chinese Medicine, 312 Anshanxi Road, Tianjin 300193, China; 3 College of Pharmacy, Tianjin Medical University, Tianjin 300070, China; 13612069165@163.com

**Keywords:** compound xueshuantong, multicomponent characterization, IM-QTOF-MS, data-dependent acquisition, automated peak annotation, component knockout

## Abstract

The state of the art ion mobility quadrupole time of flight (IM-QTOF) mass spectrometer coupled with ultra-high performance liquid chromatography (UHPLC) can offer four-dimensional information supporting the comprehensive multicomponent characterization of traditional Chinese medicine (TCM). Compound Xueshuantong Capsule (CXC) is a four-component Chinese patent medicine prescribed to treat ophthalmic disease and angina. However, research systematically elucidating its chemical composition is not available. An approach was established by integrating reversed-phase UHPLC separation, IM-QTOF-MS operating in both the negative and positive electrospray ionization modes, and a “Component Knockout” strategy. An in-house ginsenoside library and the incorporated TCM library of UNIFI^TM^ drove automated peak annotation. With the aid of 85 reference compounds, we could separate and characterize 230 components from CXC, including 155 ginsenosides, six astragalosides, 16 phenolic acids, 16 tanshinones, 13 flavonoids, six iridoids, ten phenylpropanoid, and eight others. Major components of CXC were from the monarch drug, Notoginseng Radix et Rhizoma. This study first clarifies the chemical complexity of CXC and the results obtained can assist to unveil the bioactive components and improve its quality control.

## 1. Introduction

Compound formulae of traditional Chinese medicine (TCM), rather than a single herb, have been used for thousands of years for the prevention and treatment of diseases. A key obstacle involved in the process of TCM modernization and globalization lies in the dimness of the chemical composition, which largely restricts the investigations with respect to pharmacology, mechanism of action, and quality control [1]. The combination of multiple TCM species in a formula significantly complicates the chemicals involved, featured by large spans of polarity, molecular mass, and content [2], which renders holistic evaluation of the quality rather difficult to implement for TCM formulae [3].

Advances in analytical chemistry have facilitated the comprehensive profiling and characterization of the whole metabolome from herbal medicines or TCM formulae [4]. Impressive achievements have been made in chromatography, mass spectrometry, and the data processing solutions, which drive TCM analysis towards the trends of “systematicness” and “intelligentization” [5]. On one hand, advanced column chromatography technologies and ultra-high pressure-enduring instruments greatly accelerate the efficiency of analysis and expand the coverage of analyses [6]. Particularly, the rapid development of two-dimensional liquid chromatography (2D-LC) can extend peak capacity and enhance selectivity in separation, and thus can provide deep insights into the chemical complexity of natural products [7,8]. On the other hand, hybrid high-resolution mass spectrometry, facilitates accurate MS^n^ (*n* ≥ 2) measurement, diverse scan methods, and alternative fragmentation modes [9,10]. The development of diverse data processing approaches and availability of the commercial or in-house established database can streamline the deconvolution of mass spectra and efficiently annotate the obtained high-accuracy MS^n^ data [11,12]. The MS^n^ data, used for multicomponent characterization of herbal medicines or bio-samples, are preferably acquired by data dependent acquisition (DDA) or data independent acquisition (DIA) approaches in an untargeted mode [13,14,15]. In addition, the introduction of ion mobility enables an additional dimension of separation for ions based on the charge state, size, and shape. Hybrid ion mobility/quadrupole time-of-flight mass spectrometry (IM/QTOF-MS) coupled with LC enables up to three-dimensional separations and provides four dimensions of information associated with the structures of analytes (including t_R_, drift time or collision cross section-CCS, MS^1^, and MS^2^) [16,17].

Compound Xueshuantong is a four-component formula, consisting of Notoginseng Radix et Rhizoma (San-Qi; NRR; *Panax notoginseng*), Salviae Miltiorrhizae Radix et Rhizoma (Dan-Shen; SMRR; *Salvia miltiorrhiza*), Astragali Radix (Huang-Qi; AR; *Astragalus membranaceus*), and Scrophularize Radix (Xuan-Shen; SR: *Scrophularia ningpoensis*), prescribed to treat ophthalmic disease and angina. Pharmacological research has indicated Compound Xueshuantong Capsule (CXC) could attenuate diabetic nephropathy [18] and diabetic retinopathy in rats [19] and protect blood circulation disorders [20,21]. The chemical studies regarding CXC are available, but with limitations. We consider, by conventional HPLC or LC-MS approaches, a very limited number of chemicals can be characterized. A chemical profiling research of CXC by UHPLC/Q-Orbitrap-MS operating in both the negative and positive electrospray ionization modes (ESI‒ and ESI+) identified or tentatively characterized 52 components with the aid of 21 reference compounds [22]. A liquid chromatography/tandem mass spectrometry-multiple reaction monitoring (LC-MS/MS-MRM) method has been established to quantitatively analyze eight compounds (involving harpagide, harpagoside, notoginsenoside R1, ginsenosides Rg1 and Rb1, salvianolic acid B, tanshinone IIA, and astragaloside IV) that can represent five classes of bioactive ingredients in CXC [23]. Literature with respect to the phytochemistry has informed the presence of ginsenosides from NRR [24,25,26,27], phenolic acids and terpenoids (tanshinones) from SMRR [28], triterpenoid saponins (astragalosides) and flavonoids from AR [29], and iridoids and phenylpropanoids from SR [30]. Due to the overlapping, those minor chemicals present in CXC may fail to be exposed and characterized, which necessitates the development of more potent analytical strategy to enable the comprehensive profiling and characterization of the multicomponents from CXC.

**Figure 1:**
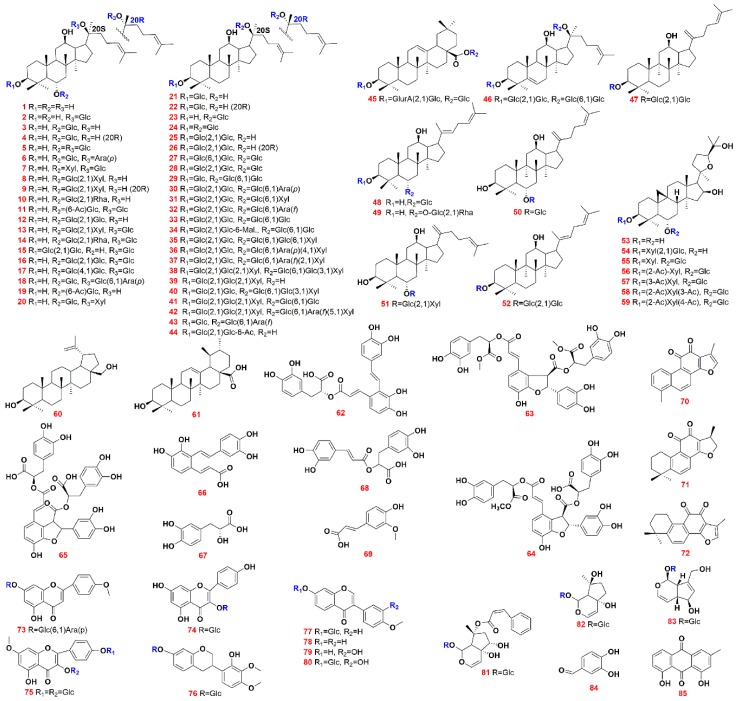
Chemical structures of 85 reference compounds used in the analysis of Compound Xueshuantong Capsule (CXC).

In the current work, a powerful platform of UHPLC/IM-QTOF-MS was utilized, aiming to boost the coverage in comprehensive profiling and characterization of the multicomponents from CXC by the following efforts: 1) integration of rapid reversed-phase UHPLC, ion mobility separation, and high-accuracy negative and positive ESI-DDA-MS^2^, to fully resolve and acquire four-dimensional information of the multicomponents (t_R_, CCS, MS^1^, and MS^2^); 2) development of a “Component Knockout” approach to enhance the exposure of much more minor components by use of the configured switching wave; 3) use of more reference compounds (Figure 1; 85 in total representative of ginsenosides, astragalosides, triterpenoids, phenolic acids, tanshinones, flavonoids, iridoids, and the others) and the reference drugs of NRR, SMRR, AR, and SR, to enhance the reference rendering the identification results more definitive; and 4) application of the powerful UNIFI^TM^ informatics software to efficiently process and automatically annotate the obtained DDA data by searching against an in-house ginsenoside library and the incorporated TCM library for SMRR, AR, and SR [17]. As a consequence, a number of components are newly characterized from CXC, which can demonstrate the potency of the currently developed approach. Hopefully, it can be an example applicable to the systematic multicomponent characterization of TCM, in particular the compound TCM formulae.

## 2. Results and Discussion

### 2.1. Optimization of a UHPLC/IM-QTOF-MS Approach Dedicated to Separating and Characterizing the Multicomponents from CXC

Prior to optimizing the chromatography condition, the extraction solvents used for sample preparation were compared. We examined five different ratios of methanol (pure water, 30% MeOH, 50% MeOH, 70% MeOH, and 100% MeOH) on the extraction efficiency of complex components from CXC. Considering that multiple classes of plant metabolites, which have been reported from CXC (e.g., ginsenosides, astragalosides, phenolic acids, flavonoids, iridoids, and phenylethanoids), could be well ionized in the negative ESI mode, and the positive mode could better ionize the less polar tanshinone analogs [22], in this work, we mainly analyzed the negative ESI-DDA-MS^2^ data, taking the positive data as a supplement. It was found that the negative ESI profiles of CXC extracted using different ratios of methanol were similar. The major chromatographic peaks eluted between 7–15 min were ginsenosides. Both the PPT-type (such as noto-R1, Rg1, and Re) and PPD-type (Rb1 and Rd) were better extracted by 70% MeOH than the other solvents, which was consistent with the tendency for some minor ginsenosides and astragalosides eluted at 16–20 min, shown in the enlarged region (Figure S1). In the case of the positive ESI mode, evaluated by tanshinones (Peaks 1–4), the solvents of 70% MeOH and MeOH were better than the others. Collectively, we considered 70% MeOH was the most suitable solvent for extracting the multicomponents from CXC.

The UHPLC condition was optimized to enable good separation of the multicomponents from CXC. Selection of the stationary phase was conducted by comparing ten UHPLC columns packed with sub-2 µm particles purchased from two major vendors (Waters Corporation and Agilent Technologies), using the number of peaks resolved from CXC and peak symmetry as the index. Considering the presence of phenolic components like salvianolic acids, flavonoids, iridoids, etc. in CXC, to maintain good peak symmetry, formic acid (FA) was employed as the additive in the water phase [22]. These candidate columns, including HSS T3, HSS C18 SB, CSH C18, BEH Shield RP18, BEH C18, CORTECS UPLC C18+, (Waters Corporation), ZORBAX Extend C18, ZORBAX SB C18, ZORBAX SB-Aq, and ZORBAX Eclipse Plus C18 (Agilent Technologies), were eluted by a unified, unoptimized gradient elution program and maintained at 35 °C. These stationary phases tested are differentiated in the silica gel core (fully porous or core-shell), the bonding technologies, and the bonding groups [15,17]. The base peak intensity (BPI) chromatograms of CXC obtained on these ten candidate columns are exhibited in Figure S2 (the left), while the peaks that could be resolved are displayed in the scatter plots (the right column). The results from the negative ESI data showed ZORBAX SB C18 (2233), CORTECS UPLC C18+ (1928), and ZORBAX Extend C18 (1732) could separate much more peaks from CXC than the other seven columns. By further comparison of these three columns, the peaks resolved by ZORBAX SB C18 were more balanced with spinous peaks, so it was, thus, selected. By further slight adjustment of the gradient eluting program, a satisfactory reversed-phase UHPLC (RP-UHPLC) approach was established (using a ZORBAX SB C18 column and 0.1% FA in acetonitrile/0.1% FA in water as the mobile phase), which could well resolve the multicomponents of CXC in 30 min.

**Figure 2:**
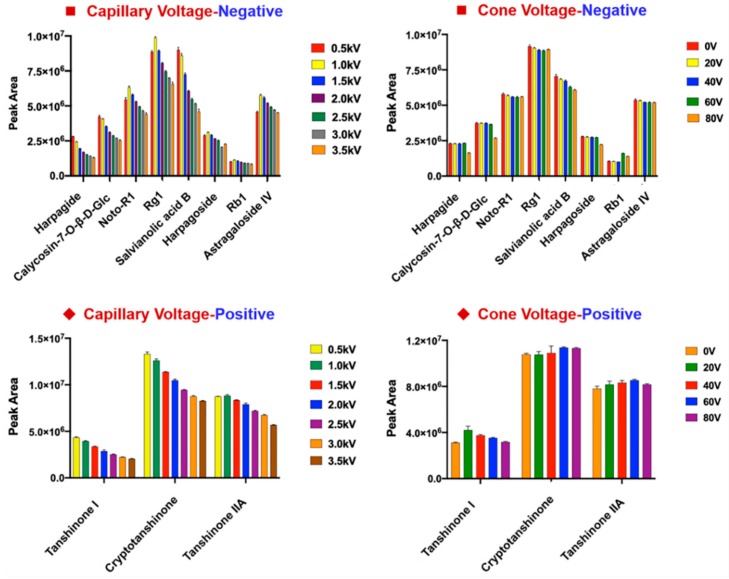
Optimization of two key ion source parameters (capillary voltage and cone voltage) of the ion IM-QTOF mass spectrometer in both the negative and positive ESI modes, to enable better characterization of the multicomponents from CXC (*n* = 3).

Key parameters of the Vion IM-QTOF instrument, which could influence the ion response and the fragmentation of CXC components in both the negative and positive ESI modes, were optimized. Two ion-source parameters, including capillary voltage (0.5–3.5 kV) and cone voltage (0–80 V), were optimized using single-factor experiments. By considering the component diversity of CXC, eleven reference compounds, involving noto-R1, Rg1, Rb1 (ginsenoside), astragaloside IV (astragaloside), salvianolic acid B (phenolic acid), calycosin-7-*O*-β-d-glucoside (flavonoid), harpagide, harpagoside (iridoid) in negative mode, and tanshinones I, -IIA, cryptotanshinone in the positive mode. Histograms showing the variation tendencies of the ion response for these eleven components among different levels of capillary voltage and cone voltage settings are shown in Figure 2. In the negative mode, five of eight index compounds were best ionized at a capillary voltage of 1.0 kV, while the other three (harpagide, calycosin-7-*O*-β-d-Glc, and salvianolic acid B) showed the highest ion response at 0.5 kV. Comparatively, the response for three saponins (noto-R1, Rg1, and astragaloside IV) at 1.0 kV was much higher than that obtained at 0.5 kV, whilst the response difference between 1.0 kV and 0.5 kV for noto-R1, Rg1, and astragaloside IV, was relatively minor. Therefore, the capillary voltage in the negative mode was set at 1.0 kV, consistent with the choice we made in the qualitative analysis of ginsenosides on this instrument [15,17]. The cone voltage, ranging from 0–80 V, in general, exerted little influence on the negative ionization of CXC multicomponents. The ion response for harpagide and calycosin-7-*O-*β-d-Glc showed a sharply decreasing trend when cone voltage increased to 80 V. However, ginsenoside Rb1 could be better ionized at the high levels of cone voltage (60 and 80 V). Collectively, we selected cone voltage of 20 V for analyzing the multicomponents from CXC. In the case of positive ESI mode mainly set for analyzing tanshinones, low levels of capillary voltage (0.5 and 1.0 kV) could benefit the ionization. Despite the fact that ion response of tanshinone I and cryptotanshinone was stronger at 0.5 kV than that obtained at 1.0 kV, the advantage was not significant. To ensure high stability and high ionization efficiency, we still selected capillary voltage at 1.0 kV in the positive mode. Cone voltage in the positive mode could bring slight effects on the ion response of tanshinones, but the tendency was different between tanshinones I and cryptotanshinone/tanshinones IIA. A low level of cone voltage at 20 V was selected in the positive ion mode. To enable sufficient fragmentation for diverse classes of components in CXC, we selected to apply mass-dependent ramp collision energy (MDRCE). In contrast to the collision energy ramp or combination of differentiated collision energy, MDRCE in DDA mode can enable customized collision energy ramp on the precursors all through the predefined scan range [17], which is more suitable for acquiring the fragmentation information of a complex chemical system consisting of diverse classes of natural compounds with large mass span, such as the TCM formulae. Using the same eleven reference compounds, the CID-MS^2^ spectra obtained at five different levels of MDRCE in the negative ESI mode were compared (low mass: 10–20 eV/high mass: 40–60 eV; 30–50 eV/50–70 eV; 40–60 eV/60–80 eV; 60–80 eV/80–100 eV; 15–30 eV/70–100 eV) (Figure S3). It was found that, the setting of 15–30 eV/70–100 eV was able to better dissociate three ginsenosides (noto-R1, Rg1, and Rb1), despite the fact that fragments of Rg1 were less rich than those obtained at 10–20 eV/40–60 eV. Similar fragmentation results were obtained for the other five representative reference compounds, yielding more balanced and diverse fragments useful for structural elucidation of iridoids, flavonoids, salvianolic acids, and astragalosides. In the positive mode using three tanshinones as the index components, another five levels of MDRCE levels were set and evaluated (10–20 eV/40–60 eV; 15–30 eV/30–45 eV; 20–30 eV/30–40 eV; 30–40 eV/40–50 eV; 40–50 eV/50–60 eV). Based on the diversity of the MS^2^ fragments obtained, we considered the MDRCE of 20–30 eV/30–40 eV more suited for dissociating tanshinones in the positive mode.

### 2.2. Development of a “Component Knockout” Approach to Enhance the Exposure of Minor Components from CXC

**Figure 3:**
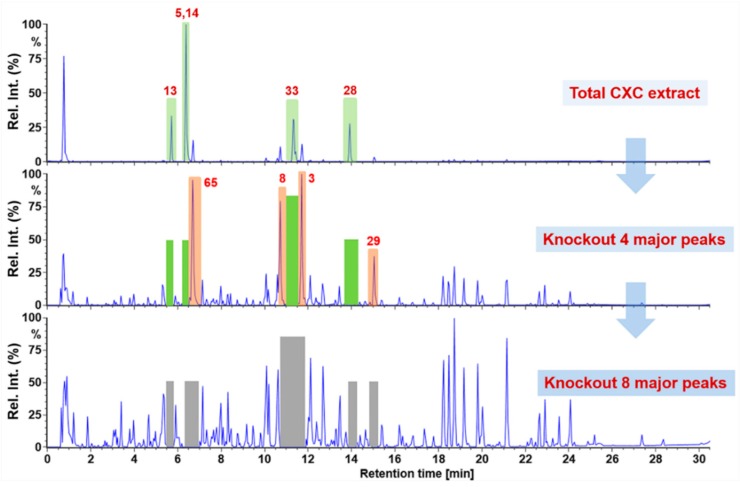
The base peak intensity chromatograms of the total CXC extract (concentration: 1 mg/mL; injection volume, 3 µL), four-peak knockout CXC (concentration: 10 mg/mL; injection volume, 3 µL), and eight-peak knockout CXC (concentration: 10 mg/mL; injection volume, 5 µL). Numbering of the components involved in these eight peaks knockout is consistent with that in Figure 1 and Table S1. **13**: noto-R1; **5**: Rg1; **14**: Re; **33**: Rb1; **28**: Rd; **65**: salvianolic acid B; **8**: noto-R2; **3**: Rh1; **29**: gypenoside XVII.

The established UHPLC/IM-QTOF-MS using DDA in both two ionization modes (ESI‒ and ESI+) was utilized to profile and characterize the multicomponents from CXC. Base peak intensity chromatogram of CXC is exhibited in Figure 3, in which less than ten major peaks were readily observed. Aiming to improve the exposure of the multicomponents from CXC, particularly those minor ones, we applied a “Component Knockout” strategy, similar to the previous report [26], by use of the automatic switching function of eluate. In detail, three injections were performed in sequence. Major components in CXC were characterized based on the total extract (concentration: 1 mg/mL; injection volume: 3 µL). Four major peaks corresponding to five compounds were switched to the waste and the CXC sample at a higher concentration (10 mg/mL) was injected in the second injection, by which much more minor peaks got enriched, as witnessed in the BPI chromatogram. In the third injection, eight major and medium peaks were removed by waste switching five times and the injection volume was increased to 5 µL, which indicated the amount of sample loaded onto the column was enhanced by 16.7 folds. By these efforts, those minor components were sufficiently exposed, which could lay a foundation for characterizing many more components from CXC. The positive results, due to the application of the knockout strategy, can be embodied in the number of the matched components by searching against the in-house ginsenoside library (for characterization of ginsenosides from NRR) and the TCM database of SMRR, AR, and SR. The “Identified Components” list (well matched with the database) of the DDA data obtained by injecting the total CXC extract was 79, and the “Unidentified Components” (the components with no matching) were 410. By applying the knockout strategy, the “Identified Components” and “Unidentified Components” significantly increased to 350 (by 4.43 folds) and 766 (by 1.87 folds), respectively. More importantly, the knockout approach was performed online (highly automatic), which has large potential for exposing and identifying more components from the complex matrix.

### 2.3. Systematic Characterization of Multiple Types of Components from CXC by Applying UNIFI^TM^ to Annotate the DDA Data Obtained in Both the Negative and Positive ESI Modes

The multicomponents from CXC were profiled by the established UHPLC/IM-QTOF-MS approach operating in both the negative and positive ESI modes, and the “Component Knockout” strategy. High-definition DDA (HD-DDA) data were acquired as well to provide CCS information for the components characterized. Automated workflows facilitated by UNIFI^TM^ were employed to efficiently interpret the obtained DDA and HD-DDA data by searching against the in-house ginsenoside library and the TCM database of SMRR, AR, and SR, in which a total of 639 compounds have been recorded (499 ginsenosides in the in-house library; 60 for AR, 58 for SMRR, and 28 for SR). Moreover, as many as 85 reference compounds (Figure 1), together with four component drugs (NRR, AR, SMRR, and SR), were used for reference to boost the reliability of the identification results and to assign the source of these identified compounds. The base peak intensity chromatograms of CXC and its component drugs are given in Figure S4. By these efforts, we could identify or tentatively characterize 230 components from CXC, which included 155 ginsenosides, six astragalosides, 16 phenolic acids, 16 tanshinones, 13 flavonoids, six iridoids, ten phenylpropanoids, and eight others. Detailed information of all these components identified from CXC is provided in Table S2.

#### 2.3.1. Characterization of Ginsenosides

The negative CID-MS^n^ features for ginsenosides are featured by the successive eliminations of the sugar moieties as neutral loss, and the generation of deprotonated sapogenin ions [13,31,32]. However, severe in-source fragmentation occurred in the positive ESI mode, yielding little diagnostic information for the structural elucidation (aside from the predominant sapogenin product ions), which would be studied in another work. Therefore, the characterization of ginsenosides from CXC was mainly based on the negative CID-MS^2^ data by applying the workflows of UNIFI and comparing with NRR and reference compounds. We could identify or tentatively characterize a total of 155 ginsenosides (accounting for 67.39% of the total amount) from CXC, which could be identified from NRR. We can draw a conclusion that the major components present in CXC are from the monarch drug NRR, evidenced from Figure 3. According to the differences on the sapogenins and acyl substitution, the ginsenosides identified can be classified into protopanaxadiol type (PPD, 44 in total), protopanaxatriol type (PPT, 52), oleanolic acid type (OA, 8), octillol type (OT, 1), malonylated (12), and the others (38). Exclusively, the neutral loss masses of the sugars eliminated from the deprotonated or FA-adduct molecules include 162.05 Da for glucose (Glc), 146.06 Da for rhamnose (Rha), 176.03 Da for glucuronic acid (GlurA), and 132.04 Da for xylose (Xyl) and arabinose (Ara) [31]. What is different, deprotonated sapogenin-related product ions are composed by *m/z* 475.38/391.29 for the PPT type, *m*/*z* 459.38/375.29 for the PPD type, and *m*/*z* 455.35 for the OA type. Moreover, typical neutral loss observed at 44.01/86.00 Da accords with the presence of single polar malonylation, while neutral loss of 88.02/172.09 Da can indicate dimalonyl substitution on the common PPD-type or PPT-type saponins [33,34]. For convenient expression, we used Xyl to represent all the possible pentose characterized by NL of 132 Da from CXC.

**Figure 4:**
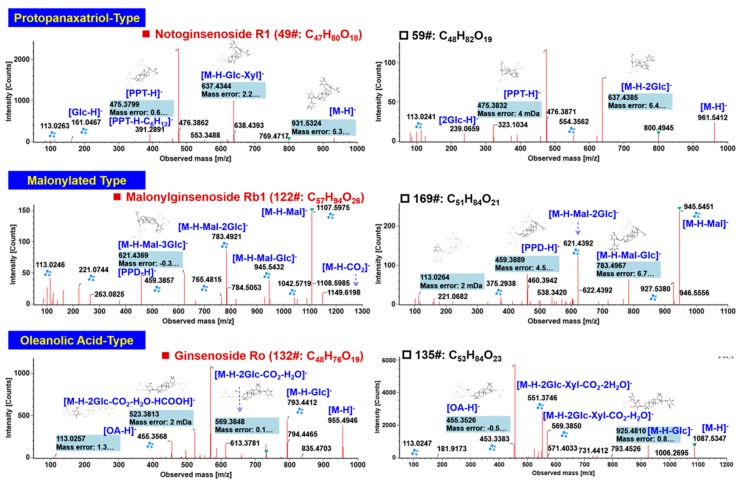
Annotation of the negative CID-MS^2^ spectra of three major subclasses of ginsenosides (protopanaxatriol-type, malonylated type, and oleanolic acid-type) identified from CXC.

The reference compound, notoginsenoside R1 (C_47_H_80_O_18_), consistent with compound 49# (t_R_ 5.74 min; Table S2), is a characteristic saponin for NRR having a sapogenin of PPT [13,26,27]. Under the current MS condition (MDRCE: 15–30 eV for low mass/70–100 eV for high mass), the negative ESI-MS/MS fragmentation of the FA-adduct precursor ion at *m*/*z* 977 could readily generate a series of abundant product ions at *m*/*z* 931.53 ([M − H]^−^), 769.47 ([M – H − Glc]^−^), 637.43 ([M − H− Glc − Xyl]^−^), 475.39 ([M − H − 2Glc − Xyl]^−^), and 391.29 (Figure 4). The secondary fragment of *m*/*z* 391.29, as a result of neutral loss of C_6_H_12_ on the C_17_-side chain, together with the sapogenin ion of *m*/*z* 475.39, can be used to differentiate the PPT sapogenin from its isomers. In the case of an unknown compound 59# (t_R_ 6.10 min, *m*/*z* 1007.5461; C_48_H_82_O_19_), the observation of characteristic product ions of *m*/*z* 475.39 and 391.29 could inform a PPT-type ginsenoside. The other product ions, dissociated from the precursor ion *m*/*z* 1007.55, including *m*/*z* 961.54, 637.44, and 475.38, could inform the presence of three Glc attached to the PPT sapogenin. Another fragment observed at *m*/*z* 323.10 corresponded to the deprotonated ion of GlcGlc. Based on this evidence, we can tentatively characterize compound 59# as PPT-(Glc − Glc)-Glc. By searching the in-house ginsenoside library, it matched seven candidate components (ginsenosides Re1–Re3, notoginsenosides R3, -R6, -M, and chikusetsusaponin LM4). Due to the unavailability of sufficient reference compounds, its structure could not be exactly identified.

The PPD- or PPT-type ginsenosides can suffer from various acyl modifications, such as malonylation. Malonylginsenosides are a characteristic subcategory of ginsenosides that are abundant in the flower buds or roots of several *Panax* species like *P. ginseng* and *P. quinquefolius* [32,33,34]. Malonylginsenosides, by negative CID-MS^2^, was featured by neutral elimination of CO_2_ even in the full-scan spectra. Neutral loss of the whole malonyl group (C_3_H_2_O_3_) occurred for the CID-MS^2^ fragmentation and a series of product ions by eliminating the sugars as those PPD- or PPT-type ginsenosides were detected. These characteristic fragmentation pathways can be evidenced from the CID-MS^2^ of a reference compound, malonylginsenoside Rb1 (compound 122#; t_R_ 11.97 min, C_57_H_94_O_26_) (Figure 4). In detail, the fragments at the high-mass region, at *m*/*z* 1149.62 and 1107.59, corresponded to the product ions after eliminating CO_2_ and the whole malonyl substituent. The other fragments were almost the same as those in the MS^2^ spectrum of Rb1. While analyzing the CID-MS^2^ spectrum of an unknown compound 169# (t_R_ 15.51 min, *m*/*z* 1031.5461; C_51_H_84_O_21_), the rich fragments at *m*/*z* 945.55, 783.49, 621.44, 459.39, and 375.29, which were highly similar to the MS^2^ fragments of Rd, could inform an isomer of malonylginsenoside Rd.

Despite the fact that OA-type ginsenosides are rare in NRR [13,27], we could identify or tentatively characterize eight ginsenosides belonging to this subtype from CXC. OA ginsenosides, in structure, are characterized by a GlurA glycosylated at 3-OH of the oleanolic acid sapogenin [25]. With regard to the CID-MS^2^ of a very common OA ginsenoside, Ro (C_48_H_76_O_19_; compound 132#, t_R_ 12.37 min), aside from the neutral cleavages of the outer sugars of two Glc (*m*/*z* 793.44 for [M − H − Glc]^−^, 613.38 for [M − H − 2Glc − H_2_O]^−^) and the observation of deprotonated OA sapogenin ion at *m*/*z* 455.36, very classic fragmentations occurred due to the presence of 28−COOH and the GlurA attached to 3-OH, with the generation of predominant product ions at *m*/*z* 569.38 ([M – H − 2Glc − H_2_O − CO_2_]^−^) and 523.38 ([M − H − 2Glc − H_2_O − CO_2_ − HCOOH]^−^) (Figure 4). These two fragments can be diagnostic to characterize OA ginsenosides bearing 3-*O*-GlurA [17,32]. An unknown saponin, compound 135# (t_R_ 12.46 min, C_53_H_84_O_23_), gave abundant deprotonated precursor ions at *m*/*z* 1087.5350. The characteristic product ions, *m*/*z* 569.39 and 455.35, could assist to characterize an OA ginsenoside. In addition, other fragments at *m*/*z* 925.48, 793.45, and 551.37 could be assigned to be [M − H − Glc]^−^, [M − H − Glc − Xyl]^−^, and [M − H − 2Glc − Xyl − CO_2_ − 2H_2_O]^−^, respectively. By comparing the CID-MS^2^ data with those of Ro, we could conclude the presence of one more pentose on Ro (or its isomer). It only matched stipuleanoside R recorded in our in-house ginsenoside library, and accordingly, we tentatively characterized compound 135# as stipuleanoside R or its isomer.

Notably, isomerism is very popular for ginsenosides in diverse *Panax* species, as demonstrated in several of our previous reports [11,13,15,17,25,31,32,34]. To more exactly assign the known ginsenosides, more dimensional structural information, such as the retention time and ion mobility-derived CCS that can be predicted by quantitative structure retention relationship (QSRR), can be obtained in our future work to enhance the reliability in ginsenoside characterization.

#### 2.3.2. Characterization of Astragalosides

**Figure 5:**
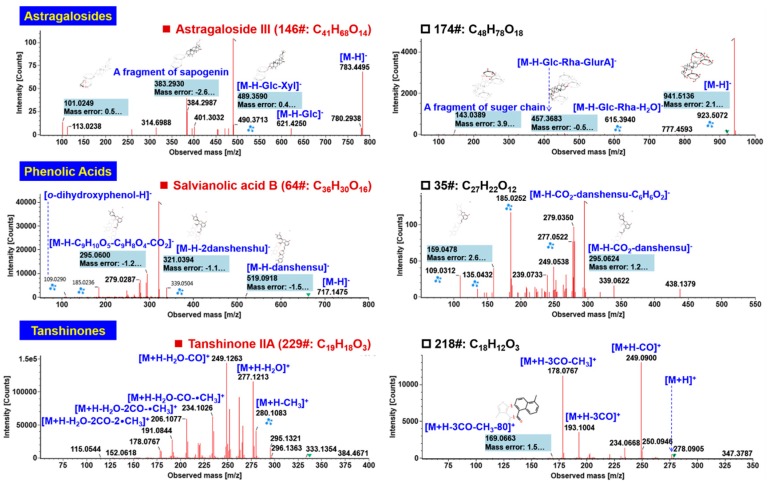
Annotation of the negative CID-MS^2^ spectra of representative components belonging to astragalosides, phenolic acids, and tanshinones identified from CXC.

Astragalosides are the major bioactive ingredients from AR [29], of which most compounds involve a cycloastragenol skeleton (C_30_H_50_O_5_) glycosylated at 3- and/or 6-OH. By analyzing the negative CID-MS^2^ data, six compounds (involving 142#, 146#, 165#, 197#, 206#, and 174#), originating from AR, were identified or tentatively characterized from CXC. Amongst them, 142# (t_R_ 12.90 min; C_41_H_68_O_14_), 146# (t_R_ 13.18 min; C_41_H_68_O_14_), 165# (t_R_ 15.26 min; C_43_H_70_O_15_), 197# (t_R_ 19.19 min; C_45_H_72_O_16_), and 206# (t_R_ 20.18 min; C_45_H_72_O_16_) were identified, with the aid of reference compounds comparison, as astragalosides IV, -III, -II, -I, and isoastragaloside I, respectively (the observed elution order was consistent with a previous report [35]). Their negative CID-MS^2^ fragmentation is featured by the easy neutral elimination of the acetyl group(s) (0–2) and sugars (Glc and Xyl), together with the generation of a sapogenin ion at *m*/*z* 489.36. However, the deprotonated cycloastragenol ion was at rather low abundance for some compounds. The CID-MS^2^ spectrum of astragaloside III (compound 146#) has been annotated as shown in Figure 5. Notably, the product ion at a medium level at *m*/*z* 383.29 should be a rearrangement product ion of the sapogenin after crossing cleavage of the furan ring. On the other hand, soyasaponins have been reported from AR as well. In the current work, we could characterize a soyasaponin, compound 174# (t_R_ 16.22 min; C_48_H_78_O_18_), from CXC. Its product ions, at *m*/*z* 941.51 ([M − H]^−^), 923.51 ([M − H − H_2_O]^−^), 615.39 ([M − H − H_2_O − Glc − Rha]^−^), and 457.37 ([M − H − Glc − Rha − GlurA]^−^; the sapogenin ion) accorded with the previous report [36], and thus we tentatively characterize compound 174# as soyasaponin I or its isomer.

#### 2.3.3. Characterization of Phenolic Acids

Phenolic acid compounds from SMRR, known as salvianolic acids [28], were characterized from CXC. Based on the negative CID-MS^2^ data, 16 phenolic acids had been identified from SMRR, of which compounds 3# (t_R_ 2.03 min, C_9_H_10_O_5_), 64# (t_R_ 6.72 min, C_36_H_30_O_16_), and 73# (tR 7.70 min, C_26_H_22_O_10_) were identified as danshensu, salvianolic acid B, and salvianolic acid A, by comparing with the reference compounds (t_R_ and MS information). The structural elucidation of a reference compound, salvianolic acid B (consistent with compound 64#: t_R_ 6.72 min, C_36_H_30_O_16_), is illustrated in Figure 5. Under the current condition, rich deprotonated precursor ion was observed at *m*/*z* 717.15 ([M − H]^−^), the CID-MS^2^ fragmentation of which could generate two abundant product ions at *m*/*z* 519.09 ([M ‒ H ‒ danshensu]^−^) and 321.04 ([M − H − 2danshensu]^−^). The fragment of *m*/*z* 339.05 could be a McLafferty rearrangement product by losing 180.04 Da (C_9_H_8_O_4_) from the fragment of *m*/*z* 519.09, which could be further fragmented into the ion *m*/*z* 295.06 by eliminating CO_2_. The weak fragment at *m*/*z* 119.03 should be ascribed to deprotonated *O*-dihydroxyphenol (C_6_H_5_O_2_^−^). These characteristic fragmentation features can assist to identify an unknown compound 35# (t_R_ 4.96 min, C_27_H_22_O_12_), giving deprotonated precursor ion at *m*/*z* 537.1048. Its CID-MS^2^ spectrum displayed some fragments similar to those of salvianolic acid B, including *m*/*z* 295.06, 279.04, 185.03, 159.05, and 109.03. Based on the difference in the molecular formulae between compound 35# and salvianolic acid B, we could deduce compound 35# had one danshensu unit, tentatively characterized as lithospermic acid or its isomer.

#### 2.3.4. Characterization of Tanshinones

Apart from salvianolic acids, the non-polar tanshinones are another category of bioactive ingredients in SMRR [28]. A total of 16 tanshinone components were identified or tentatively characterized from CXC based on the analysis of the positive CID-MS^2^ data, of which compounds 221# (t_R_ 24.14 min; C_18_H_12_O_3_), 223# (t_R_ 24.21 min; C_19_H_12_O_3_), and 229# (t_R_ 25.96 min; C_19_H_18_O_3_) were identified, by comparing the reference compounds, as tanshinone I, cryptotanshinone, and tanshinone IIA, respectively. Annotation of the positive CID-MS^2^ spectrum of the reference compound tanshinones IIA (229#) is illustrated in Figure 5. Diverse product ions, as the result of radical cleavages of •CH_3_ and neutral loss of H_2_O and CO were readily dissociated from the protonated precursor ion at *m*/*z* 295.13 [37]. The unknown tanshinones compound 218# (t_R_ 23.57 min; C_18_H_12_O_3_) was characterized as an isomer of tanshinones I, as similar fragmentation pathways were observed (Figure 5).

#### 2.3.5. Characterization of Flavonoids

**Figure 6:**
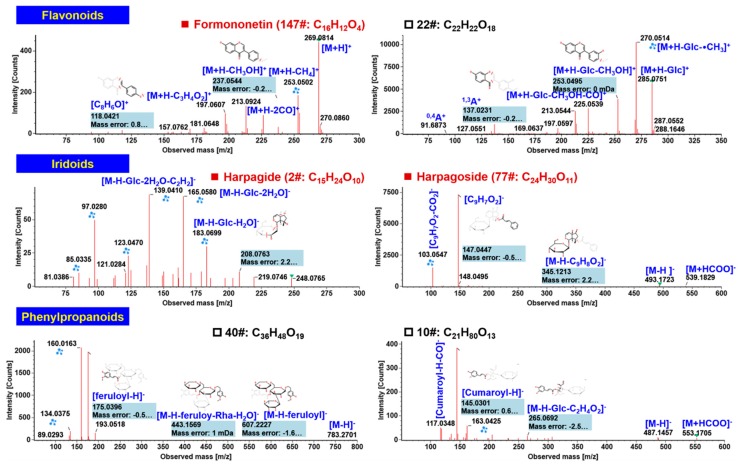
Annotation of the negative CID-MS^2^ spectra of representative components belonging to flavonoids, iridoids, and phenylpropanoids identified from CXC.

Flavonoids are a class of widely distributed bioactive components for plants. AR contains rich isoflavonoids [29]. We could characterize 13 flavonoids, including two free flavonols (compounds 19# and 37#) and the *O*-glycoside (17#) from NRR, and ten isoflavones (21#, 22#, 23#, 38#, 39#, 71#, 74#, 80#, 147#, and 230#) from AR. Characteristic fragmentations for isoflavones in the positive mode could be evidenced from the CID-MS^2^ spectrum of the reference compound, formononetin (147#; C_16_H_12_O_4_), as shown in Figure 6. Rich fragments were dissociated from the protonated precursor at *m*/*z* 269.08 ([M + H]^+^), such as *m*/*z* 253.05 ([M + H − CH_4_]+), 213.00 ([M + H − 2CO]^+^), 237.05 ([M + H − CH_3_OH]^+^), 197.06 ([M + H − C_3_H_4_O_2_]^+^), and 118.04 (C_8_H_8_O^+^]). The unknown compound 22# (t_R_ 3.80 min; C_22_H_22_O_10_) was characterized as a glycosidic isoflavone. Protonated precursor at *m*/*z* 447.13 could readily eliminate the attached Glc generating the aglycone ion of *m*/*z* 285.07. The other fragments of the aglycone moiety, such as *m*/*z* 270.06 ([M + H − Glc − ^•^CH^3^]^+^), 253.05 ([M + H − CH_3_OH]^+^), 225.05 ([M + H − CH_3_OH − CO]^+^), 137.02 (^1,3^A^+^), and 91.59 (^0,4^A^+^), could inform an additional hydroxyl on ring B other than formononetin. Accordingly, this compound was tentatively characterized as campanulin or its isomer [22].

#### 2.3.6. Characterization of Iridoids

Iridoids are one of the main constituents of SR [30] and the basic parent nucleus of iridoids is cycloallyl ether terpene, which involves cycloallyl ether and alcohol hydroxyl groups. Since the alcohol hydroxyl group belongs to hemiacetal hydroxyl group and has active properties, most of these compounds are in the form of glycosides. From the point of molecular structure, the basic framework of iridoid glycosides contains a characteristic dihydropyrane ring, which concatenates a five-membered ring unit structure. According to the structure of the terpene glycoside parent nucleus, it can be divided into cyclopentene type, cyclopentane type, and epoxide type. We could characterize a total of six iridoids components from CXC, which all could be identified from SR. Among them, 1# (t_R_ 1.62 min, C_15_H_22_O_9_), 2# (t_R_ 1.87 min, C_15_H_24_O_10_), and 77# (t_R_ 7.98 min, C_24_H_30_O_11_) were identified, by comparing with the reference compounds, as aucubin, harpagide, and harpagoside, respectively. We illustrated the annotation of reference compounds harpagide and harpagoside in Figure 6.

#### 2.3.7. Characterization of Phenylpropanoids

Phenylpropanoids are another class of bioactive components reported from SR, in addition to iridoids [30]. The phenylpropanoids in SR consist of α,β-unsaturated phenylpropionic acid and the sugar chain. The characterization of phenylpropanoids from CXC was mainly based on the negative DDA-MS^2^ data. In our work, ten phenylpropanoids originating from SR were tentatively characterized (5#, 6#, 10#, 12#, 24#, 26#, 27#, 29#, 40#, and 44#). The structural elucidations of two unknown phenylpropanoids, 40# (t_R_ 5.26 min, *m*/*z* 783.2737 for [M − H]^−^) and 10# (t_R_ 2.82 min, *m*/*z* 487.1458) are illustrated in Figure 6. The MS/MS fragmentation of compound 40# generated a predominant product ion at *m*/*z* 607.2227 ([M − H − feruloyl]^−^), 443.1569 ([M − H − feruloyl − Rha − H_2_O]^−^), and 175.0396 (feruloyl^−^). We tentatively characterized this compound as angoroside C or its isomer by comparing with the data in literature [38]. In the case of compound 10#, diverse product ions at *m*/*z* 487.18 ([M − H]^−^), 265.07 ([M − H − Glc − C_2_H_4_O_2_]^−^), and 145.03 (cross fragmentation of cumaric acid), were generated. These product ions were similar to those of 6-*O*-*p*-coumaroylsucrose [38].

**Figure 7:**
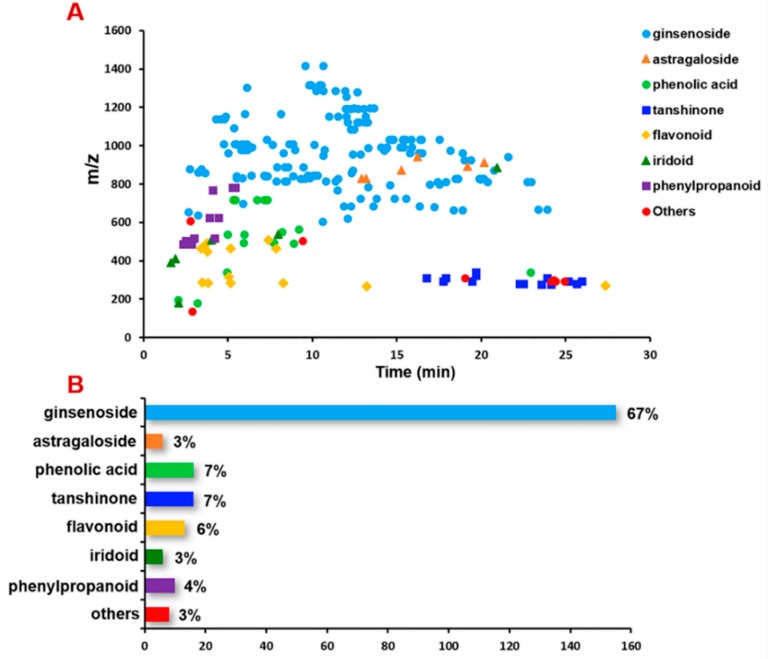
Summary of the different classes of components characterized from CXC by a 2D t_R_-*m*/*z* scatter plot (**A**) and a distribution percent histogram (**B**).

In addition, eight other compounds (9#, 11#, 93#, 196#, 222#, 225#, 226#, and 227#), and their information are given in Table S2. We gave a brief summary on the components identified from CXC. The components we have characterized remarkably differ in structure categories. The plant species present in CXC have been injected in separately, in order for the association made between the chemical constituents and the plant species that make up the capsule to be conclusive. From the distribution percent histogram, ginsenosides, assigned from the monarch drug NRR, occupy a large proportion, up to 67%. The phenolic acids (salvianolic acid analogs) and tanshinones from SMRR are ranked the second most, equal to 7% of the total amount, while flavonoids (6%), mainly from AR and NRR, are third most (Figure 7). On the other hand, a 2D scatter plot of all the components characterized from CXC has been depicted with t_R_ VS *m*/*z*, in which different classes of CXC components display certain features. Most ginsenosides almost scatter, covering the whole elution gradient, and are located in the upper region. It informs a large polarity span and relatively large molecular mass. Comparatively, tanshinones are less polar, showing longer retention time than most of the other components. Iridoids, phenolic acids, and phenylpropanoids are polar structures with t_R_ < 10 min under the current chromatographic condition. These characteristics delineate a global structural feature demonstrating the chemical complexity for CXC.

## 3. Materials and Methods

### 3.1. Reagents and Chemicals

In total, 85 compounds, involving 52 ginsenosides, seven astragalosides, two triterpenoids, eight phenolic acids, three tanshinones, eight flavonoids, three iridoids, and two others, purchased from Shanghai Standard Biotech. Co., Ltd. (Shanghai, China) or Chengdu Desite Biotechnology Co., Ltd. (Chengdu, China), were used as the reference compounds in the current work. Their chemical structures and detailed information are given in Figure 1 and Table S1, respectively. Acetonitrile, methanol (Fisher, Fair lawn, NJ, USA), and formic acid (FA; Sigma-Aldrich, St. Louis, MO, Switzerland), were LC-MS grade. Ultra-pure water was in-house prepared using a Milli-Q water purification system (Millipore, Bedford, MA, USA). The sample of Xueshuantong Capsule analyzed in this work was purchased from Guangdong Zhongsheng Pharmaceutical Co., Ltd. (Batch No. Z20030017). The samples of Astragali Radix (AR), Salviae Miltiorrhizae Radix et Rhizoma (SMRR), and Scrophularize Radix (SR) were purchased from Sichuan and Notoginseng Radix et Rhizoma (NRR) from Yunnan. All samples were deposited in the authors’ laboratory, Tianjin University of Traditional Chinese Medicine (Tianjin, China).

### 3.2. Sample Preparation

The contents of 10 capsules of CXC were mixed. The accurately weighed powder for each of CXC, NRR, AR, SMRR, and SR (100 mg) were dispersed in 10 mL 70% aqueous methanol (*v*/*v*) and vortexed for 2 min. Samples were extracted in a water bath at 40 °C assisted with the ultrasound for 40 min. The obtained liquid was transformed into a 10 mL volumetric flask, compensated with 70% methanol to the volume. Each sample was centrifuged at a rotation speed of 14,000 rpm for 10 min, leading to the supernatant used as the test solution for CXC (CXC-1) and four component drugs (NRR, AR, SMRR, and SR; 10 mg/mL). Another CXC sample (CXC-2) was prepared from the stock solution by diluting with 70% aqueous methanol for ten folds (1 mg/mL). Reference compounds were dissolved in 70% or pure methanol (approximately 1 mg/mL for each stock solution), and their solutions were prepared as mixed reference compounds solutions for LC-MS analysis.

### 3.3. UHPLC/IM-QTOF-MS

The high-accuracy MS data for the multicomponent profiling and characterization of CXC were acquired by use of an ACQUITY UPLC I-Class/Vion IMS-QTOF system (Waters Corporation, Milford, MA, USA). A ZORBAX SB C18 column (2.1 × 100 mm, 1.8 μm; Agilent Technologies, Santa Clara, CA, USA) maintained at 30 °C was used for chromatographic separation. A binary mobile phase, composed by 0.1% FA in CH3CN (organic phase: A) and 0.1% FA in H_2_O (water phase: B), was employed at a flow rate of 0.3 mL/min following an optimal gradient program: 0–2 min, 5–20% (A); 2–4 min, 20–23% (A); 4–8 min, 23–30% (A); 8–9.5 min, 30–32% (A); 9.5–16 min, 32–40% (A); 16–20 min, 40–50% (A); 20–22 min, 50%–60% (A); 22–26 min, 60–85% (A); 26–29 min, 85% (A); 29–30 min, 85–95% (A); and 30–34 min, 95% (A). A 5 min re-equilibration time was set between the successive injections.

A knockout strategy, by use of the switching wave of the Vion IMS-QTOF instrument, was applied, aiming to enhance the exposure and characterization of much more minor components in the negative ESI mode. Three injections were performed. Injection I: CXC-2 (1 mg/mL), 3 µL; Injection II: CXC-1 (10 mg/mL), 3 µL; Injection III: CXC-1 (10 mg/mL), 5 µL. In the second injection, the elutes for four peaks, at 5.5–5.8 min, 6.2–6.5 min, 11–11.5 min, and 13.8–14.1 min, were automatedly switched to the waste; for the third injection, the elutes at 5.5–5.8 min, 6.2–7.0 min, 10.7–11.9 min, 13.8–14.2 min, and 14.9–15.3 min, consistent with eight peaks, were not delivered into the mass spectrometer.

High-accuracy MS data were recorded on a Vion IMS-QTOF mass spectrometer in both the negative and positive DDA (MS-MSMS) modes (Waters Corporation, Milford, MA, USA). The LockSpray ion source was equipped using the following parameters: capillary voltage, −1.0 kV/1.0 kV; cone voltage, 20 V; source offset, 80 V; source temperature, 120 °C; desolvation gas temperature, 500 °C; desolvation gas flow (N_2_), 800 L/h; and cone gas flow (N_2_), 50 L/h. Default parameters were defined for the travelling wave IM separation. The QTOF analyzer scanned over a mass range of *m*/*z* 80−1500 at a low energy of 6 eV for both ESI modes at 0.3 s per scan (MS^1^). In DDA settings, when the intensity exceeded 1000 detector counts, MS/MS fragmentation of the five most intense precursors was automatedly triggered. Mass dependent ramp collision energy (MDRCE) of 15–30 eV in low mass ramp/70–100 eV in high mass ramp and 20–30 eV in low mass ramp/30–40 eV in high mass ramp were set for ESI−/DDA and ESI+/DDA, respectively. The MS/MS acquisition stopped if either TIC (total ion chromatogram) exceeded 2000 detector counts or time exceeded 1.5 s. High-definition DDA, by enabling the IM function, was also set to profile the components of CXC generating the CCS information, in which the scan range and scan time were in accordance with the settings for DDA. MS data calibration was conducted by constantly infusing the leucine enkephalin (LE; Sigma-Aldrich, St. Louis, MO, USA; 200 ng/mL) at a flow rate of 10 µL/min [15,17]. Calibration of CCS was conducted according to the manufacture’s guideline using a mixture of calibrants [39].

### 3.4. Date Processing

Uncorrected DDA and HDDA data in continuum format were processed by using the UNIFI 1.9.3.0 software (Waters, Milford, MA, USA). The UNIFI software performed data correction, peak picking, and peak annotation by searching against an in-house ginsenoside library and the incorporated TCM libraries of SMRR, AR, and SR. Key parameters of UNIFI are depicted as follows. Find DDA masses: MS ion intensity threshold, 100.0 counts; MSMS ion intensity threshold, 5.0 counts. Target by mass: target match tolerance, 10.0 ppm; screen on all isotopes in a candidate, generate predicted fragments from structure, and look for in-source fragments were enabled; fragment match tolerance, 10.0 ppm. Adducts: negative adducts including +HCOO, -H, +CH_3_COO, +Cl, +e. Lock Mass: combine width, 3 scans; mass window, 0.5 *m*/*z*; reference mass, 554.2620; reference charge, ‒1. Positive adducts including +H, +Na, -e. Lock mass: combine width, 3 scans; mass window, 0.5 *m*/*z*; reference mass, 556.2766; reference charge, +1.

## 4. Conclusions

Aiming to enhance the exposure and characterization of more minor components from the TCM formula CXC, in the current work, an integral approach, by combining the powerful platform of UHPLC/IM-QTOF-MS, a “Component Knockout” strategy, and the versatile data processing software UNIFI, was established. Compared with the literature [22], 4.4-fold of components were characterized by our strategy and the newly characterized components mainly involve ginsenosides. The data obtained provide new insight into the chemical complexity of CXC, which would be beneficial to the subsequent pharmacological and quality control researches.

## Supplementary Materials

The following are available online: Figure S1, Comparison of the extraction solvent (different ratios of methanol: 0, 30%, 50%, 70%, and 100%) for Compound Xueshuantong Capsule (CXC); Figure S2, Selection of the stationary phase for establishing the RP-UHPLC approach enabling good separation of the multicomponents from Compound Xueshuantong Capsule (CXC); Figure S3, Optimization of mass-dependent ramp collision energy (MDRCE) for the DDA approach established both in the negative and positive ESI modes using representative reference compounds; Figure S4, Base peak intensity chromatograms of CXC and four component TCM drugs (NRR, AR, SMRR, and SR) obtained on the Vion IM-QTOF mass spectrometer in both the negative and positive ESI modes; Table S1, Information of 85 reference compounds used in this work. The numbering was consistent with that in Figure 1; Table S2, Information of the 230 components characterized from CXC.
