# Supplementary material for: Data-Dependent Acquisition and Database-Driven Efficient Peak Annotation for the Comprehensive Profiling and Characterization of the Multicomponents from Compound Xueshuantong Capsule by UHPLC/IM-QTOF-MS
Source: Molecules. 2019 Sep 21;24(19):3431. doi: 10.3390/molecules24193431 (PMC6804152; doi:10.3390/molecules24193431)
Supplement: Supplementary file 1 [file molecules-24-03431-s001.pdf]

<**Molecules**>

Electronic Supplementary Materials

Data-dependent Acquisition and Database-driven Efficient Peak Annotation for the Comprehensive Profiling and Characterization of the Multicomponents from Compound Xueshuantong Capsule by UHPLC/IM-QTOF-MS

Tiantian Zuo [1,2,†], Yuexin Qian [1,2,†], Chunxia Zhang [1,2], Yuxi Wei [3], Xiaoyan Wang [1,2], Hongda Wang [1,2], Ying Hu [1,2], Weiwei Li [1,2], Xiaohui Wu [3,*], Wenzhi Yang [1,2,*]

[1] Tianjin State Key Laboratory of Modern Chinese Medicine, Tianjin University of Traditional Chinese Medicine, 312 Anshanxi Road, Tianjin 300193, China

[2] Tianjin Key Laboratory of TCM Chemistry and Analysis, Tianjin University of Traditional Chinese Medicine, 312 Anshanxi Road, Tianjin 300193, China

[3] College of Pharmacy, Tianjin Medical University, Tianjin 300070, China

[*] Correspondence: longhui804@163.com (X.W.); wzyang0504@tjutcm.edu.cn, Tel.: +86-022-5979-1833 (W.Y.)

[†] These two authors contributed equally to this work.

Contents

Figure S1 Comparison of the extraction solvent (different ratios of methanol: 0, 30%, 50%, 70%, and 100%) for Compound Xueshuantong Capsule (CXC). Base peak intensity chromatograms were acquired using acetonitrile/0.1% formic acid in H_2O as the mobile phase and an HSS T3 column maintained at 35°C. Peaks 1–4 in the positive ESI mode are tanshinones.

Figure S2 Selection of the stationary phase for establishing the RP-UHPLC approach enabling good separation of the multicomponents from Compound Xueshuantong Capsule (CXC). The left shows the base peak intensity (BPI) chromatograms of CXC obtained by using ten candidate columns; the right is the scatter plot of the components resolved by each candidate column.

Figure S3 Optimization of mass-dependent ramp collision energy (MDRCE) for the DDA approach established both in the negative and positive ESI modes using representative reference compounds. (**A**) Comparison in the negative mode using three ginsenoside compounds; (**B**) comparison in the negative mode using representative compounds of iridoids, flavonoids, salvianolic acids, and astragalosides; (**C**) comparison in the positive mode using three tanshinones compounds.

Figure S4 Base peak intensity chromatograms of CXC and four component TCM drugs (NRR, AR, SMRR, and SR) obtained on the Vion IM-QTOF mass spectrometer in both the negative and positive ESI modes.

Table S1 Information of 85 reference compounds used in this work. The numbering was consistent with that in Figure 1.

Table S2 Information of the 230 components characterized from CXC.

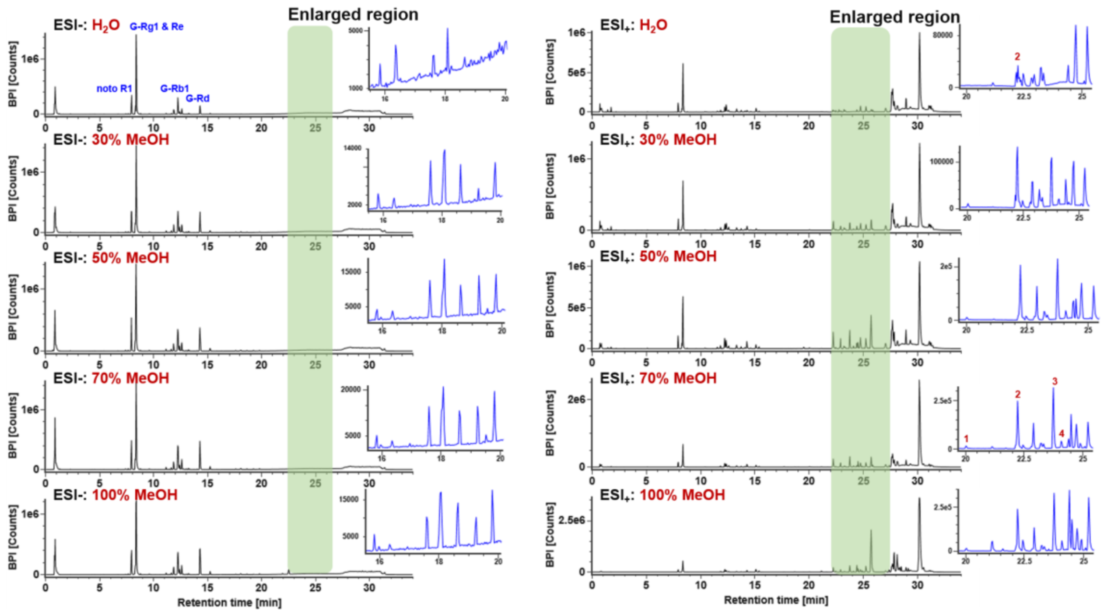

Figure S1 Comparison of the extraction solvent (different ratios of methanol: 0, 30%, 50%, 70%, and 100%) for Compound Xueshuantong Capsule (CXC). Base peak intensity chromatograms were acquired using acetonitrile/0.1% formic acid in H_2O as the mobile phase and an HSS T3 column maintained at 35°C. Peaks 1–4 in the positive ESI mode are tanshinones.

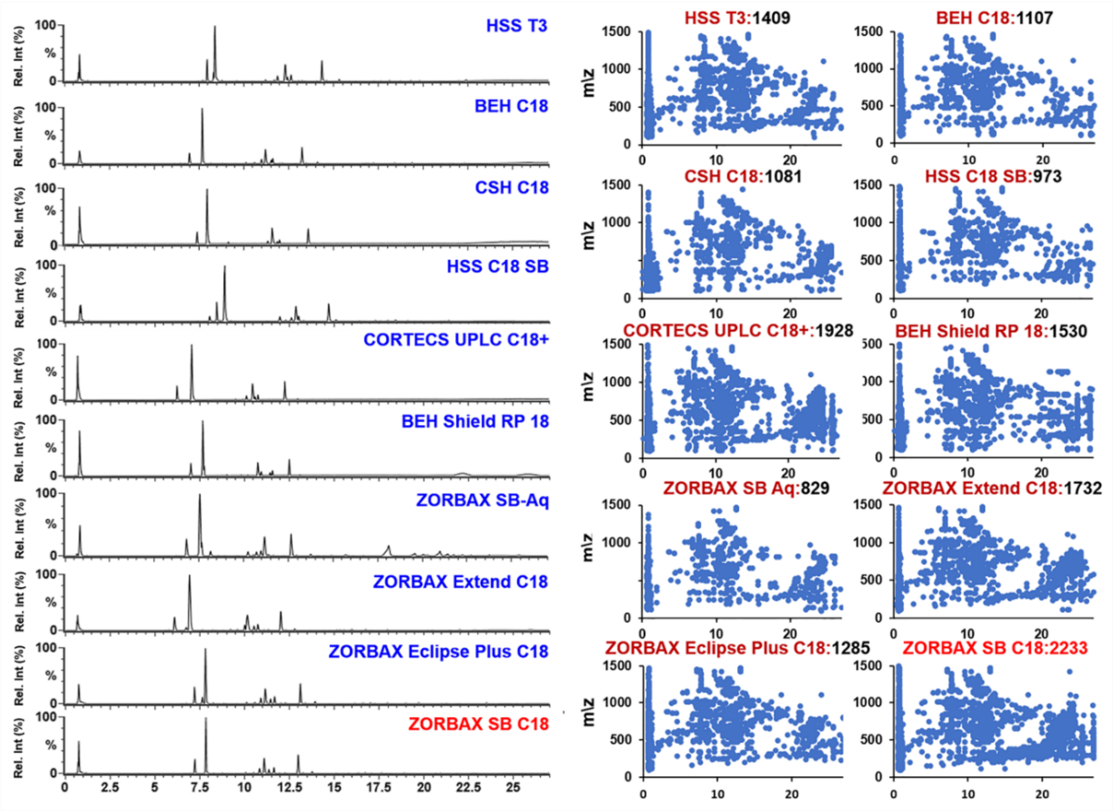

Figure S2 Selection of the stationary phase for establishing the RP-UHPLC approach enabling good separation of the multicomponents from Compound Xueshuantong Capsule (CXC). The left shows the base peak intensity (BPI) chromatograms of CXC obtained by using ten candidate columns; the right is the scatter plot of the components resolved by each candidate column.

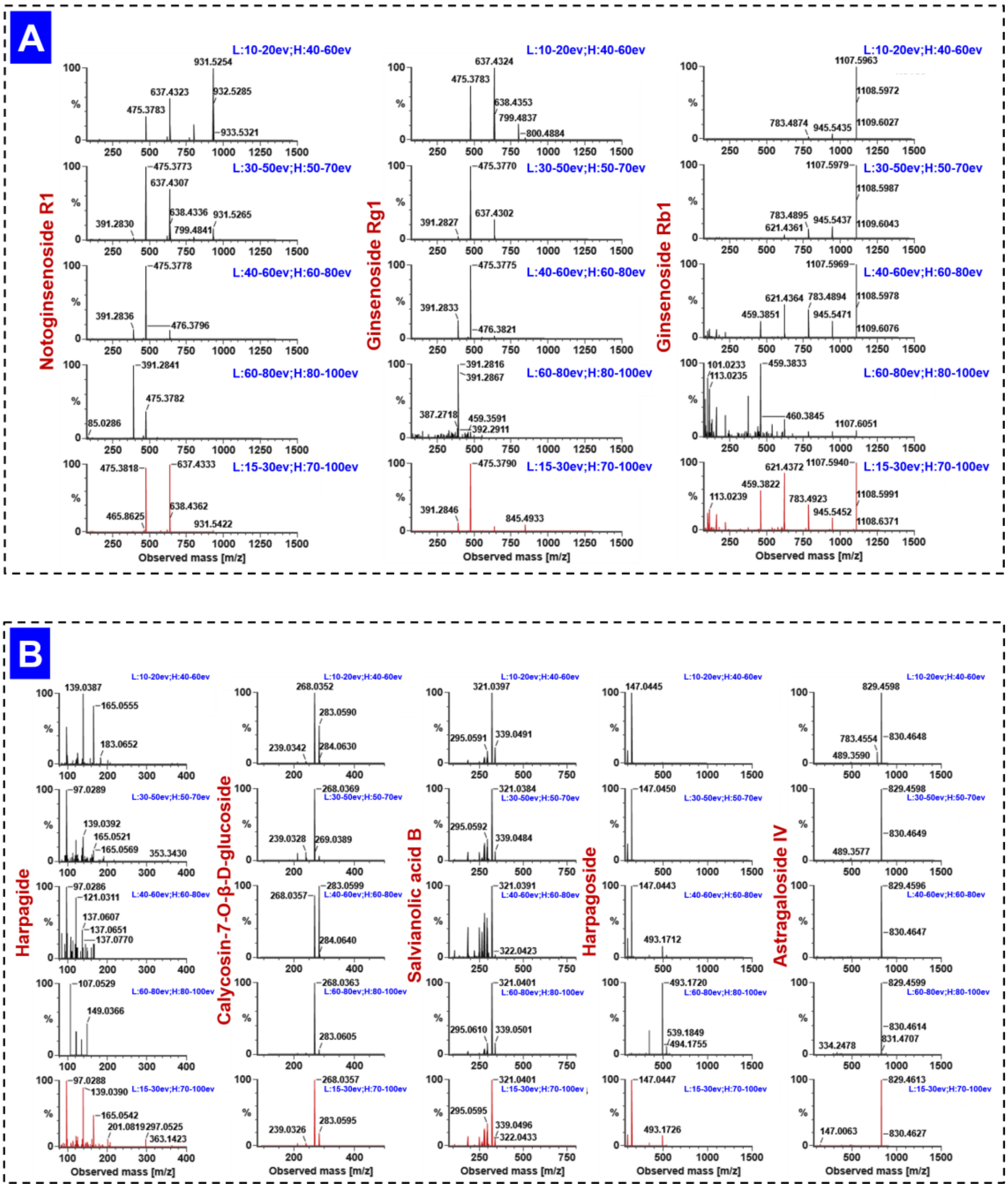
A
Notoginsenoside R1
Ginsenoside Rg1
Ginsenoside Rb1
L:10-20ev;H:40-60ev
L:30-50ev;H:50-70ev
L:40-60ev;H:60-80ev
L:60-80ev;H:80-100ev
L:15-30ev;H:70-100ev
Observed mass [m/z]
B
Harpagide
Calycosin-7-O-β-D-glucoside
Salvianolic acid B
Harpagoside
Astragaloside IV
Observed mass [m/z]

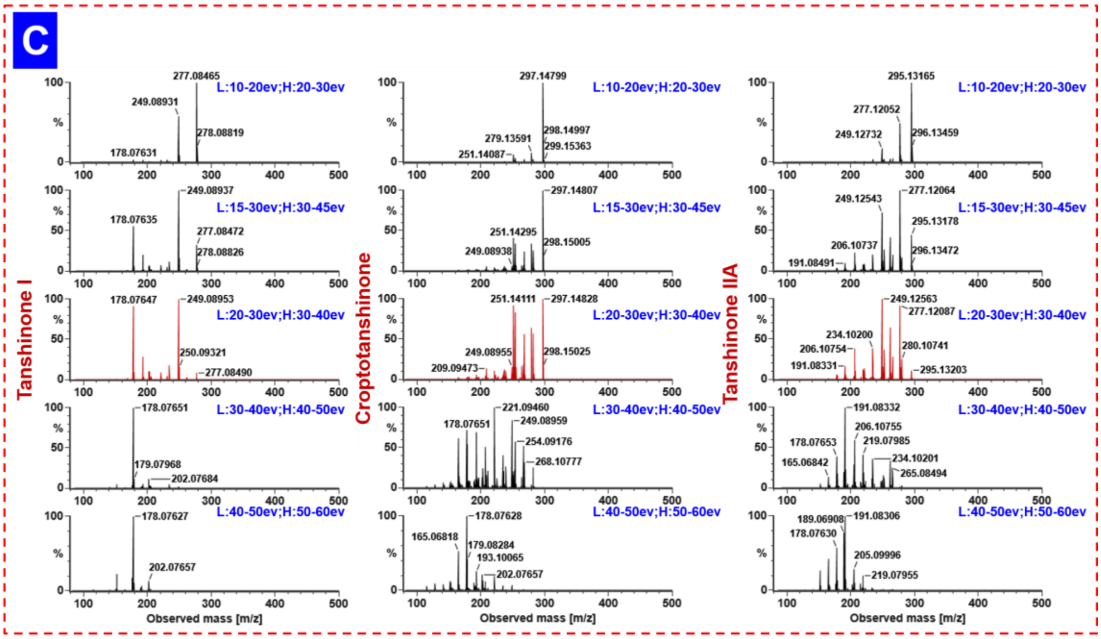

Figure S3 Optimization of mass-dependent ramp collision energy (MDRCE) for the DDA approach established both in the negative and positive ESI modes using representative reference compounds. (**A**) Comparison in the negative mode using three ginsenoside compounds; (**B**) comparison in the negative mode using representative compounds of iridoids, flavonoids, salvianolic acids, and astragalosides; (**C**) comparison in the positive mode using three tanshinones compounds.

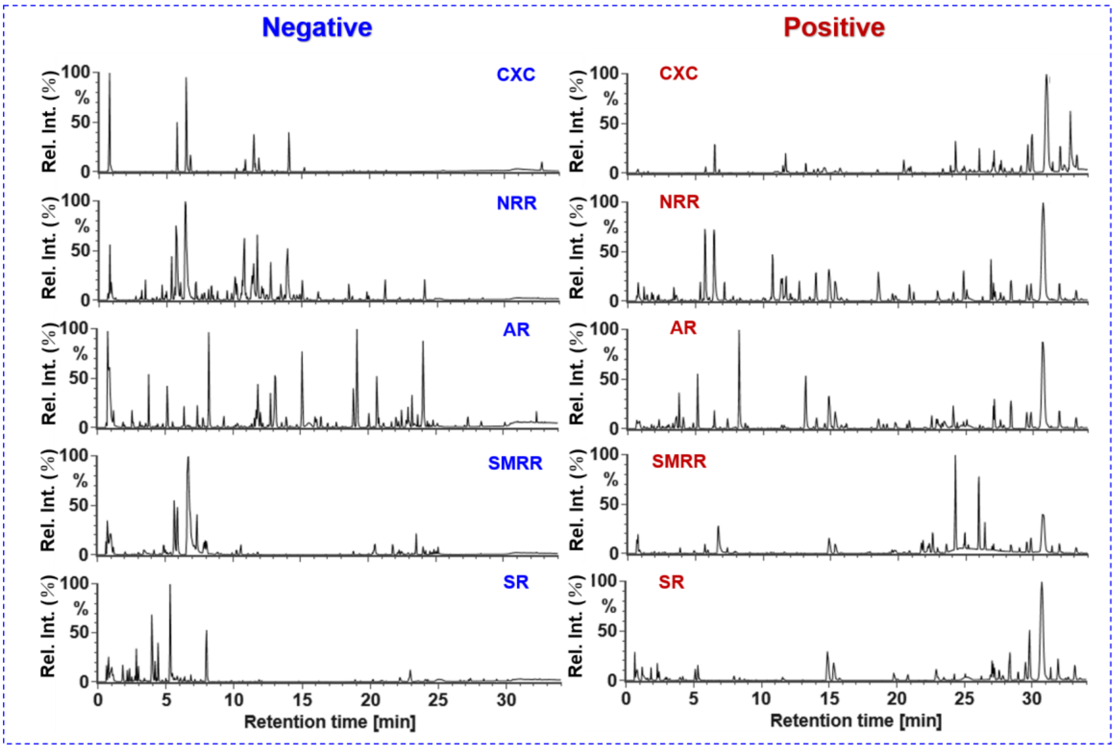

Figure S4 Base peak intensity chromatograms of CXC and four component TCM drugs (NRR, AR, SMRR, and SR) obtained in both the negative and positive ESI modes.

Table S1 Information of 85 reference compounds used in this work. The numbering was consistent with that in Figure 1.

No.	Compound	M.F.	Exact mass
**Ginsenosides**			
1	20(*S*)-protopanaxatriol	$C_{30}H_{52}O_4$	476.3866
2	ginsenoside F1	$C_{36}H_{62}O_9$	638.4394
3	ginsenoside Rh1	$C_{36}H_{62}O_9$	638.4394
4	20(R)-ginsenoside Rh1	$C_{36}H_{62}O_9$	638.4394
5	ginsenoside Rg1	$C_{42}H_{72}O_{14}$	800.4922
6	sanchinoside A3	$C_{41}H_{70}O_{13}$	770.4816
7	pseudoginsenoside Rt3	$C_{41}H_{70}O_{13}$	770.4816
8	notoginsenoside R2	$C_{41}H_{70}O_{13}$	770.4816
9	20(*R*)-notoginsenoside R2	$C_{41}H_{70}O_{13}$	770.4816
10	ginsenoside Rg2	$C_{42}H_{72}O_{13}$	784.4973
11	notoginsenoside Rt	$C_{44}H_{74}O_{15}$	842.5028
12	ginsenoside Rf	$C_{42}H_{72}O_{14}$	800.4922
13	notoginsenoside R1	$C_{47}H_{80}O_{18}$	932.5345
14	ginsenoside Re	$C_{48}H_{82}O_{18}$	946.5501
15	vinaginsenoside R4	$C_{48}H_{82}O_{19}$	962.5450
16	20-*O*-glucosylginsenoside Rf	$C_{48}H_{82}O_{19}$	962.5450
17	notoginsenoside N	$C_{48}H_{82}O_{19}$	962.5450
18	notoginsenoside Fp1	$C_{47}H_{80}O_{18}$	932.5345
19	6-O-β-D-(6'-acetyl)-glucopyranosyl-24-en-dammar-3β,6α, 12β,20S-tetraol	$C_{38}H_{64}O_{10}$	680.4499
20	6-O-(β-D-glucopyranosyl)-20-O-(β-D-xylopyranosyl)-3β,6 α,12β,20(S)-tetrahydroxy dammar-24-ene	$C_{41}H_{70}O_{13}$	770.4816
21	ginsenoside Rh2	$C_{36}H_{62}O_8$	622.4445
22	20(*R*)-ginsenoside Rh2	$C_{36}H_{62}O_8$	622.4445
23	compound K	$C_{36}H_{62}O_8$	622.4445
24	ginsenoside F2	$C_{42}H_{72}O_{13}$	784.4973
25	20(*S*)-ginsenoside Rg3	$C_{42}H_{72}O_{13}$	784.4973
26	20(*R*)-ginsenoside Rg3	$C_{42}H_{72}O_{13}$	784.4973
27	notoginsenoside K	$C_{48}H_{82}O_{18}$	946.5501
28	ginsenoside Rd	$C_{48}H_{82}O_{18}$	946.5501
29	gypenoside XVII	$C_{48}H_{82}O_{18}$	946.5501
30	ginsenoside Rb2	$C_{53}H_{90}O_{22}$	1078.5924
31	ginsenoside Rb3	$C_{53}H_{90}O_{22}$	1078.5924
32	ginsenoside Rc	$C_{53}H_{90}O_{22}$	1078.5924
33	ginsenoside Rb1	$C_{54}H_{92}O_{23}$	1108.6029
34	malonyl-ginsenoside Rb1	$C_{57}H_{94}O_{26}$	1194.6033
35	notoginsenoside R4	$C_{59}H_{100}O_{27}$	1240.6452
36	ginsenoside Ra1	$C_{58}H_{98}O_{26}$	1210.6346

37	ginsenoside Ra2	$C_{58}H_{98}O_{26}$	1210.6346
38	notoginsenoside T	$C_{64}H_{108}O_{31}$	1372.6875
39	notoginsenoside Ft1	$C_{47}H_{80}O_{17}$	916.5396
40	ginsenoside Ra3	$C_{59}H_{100}O_{27}$	1240.6452
41	notoginsenoside Fa	$C_{59}H_{100}O_{28}$	1256.6401
42	notoginsenoside S	$C_{63}H_{106}O_{30}$	1342.6769
43	notoginsenoside Fe	$C_{47}H_{80}O_{17}$	916.5396
44	ginsenoside Rs3	$C_{44}H_{74}O_{14}$	826.5079
45	ginsenoside Ro	$C_{48}H_{76}O_{19}$	956.4981
46	5,6-didehydroginsenoside Rb1	$C_{54}H_{90}O_{23}$	1106.5873
47	ginsenoside Rk1	$C_{42}H_{70}O_{12}$	766.4868
48	ginsenoside Rh4	$C_{36}H_{60}O_{8}$	620.4288
49	ginsenoside F4	$C_{42}H_{70}O_{12}$	766.4867
50	ginsenoside Rk3	$C_{36}H_{60}O_{8}$	620.4288
51	notoginsenoside T5	$C_{41}H_{68}O_{12}$	752.4711
52	ginsenoside Rg5	$C_{42}H_{70}O_{12}$	766.4868
Astragalosides			
53	cycloastragenol	$C_{30}H_{50}O_{5}$	490.3658
54	astragaloside III	$C_{41}H_{68}O_{14}$	784.4609
55	astragaloside IV	$C_{41}H_{68}O_{14}$	784.4609
56	astragaloside II	$C_{43}H_{70}O_{15}$	826.4715
57	isoastragaloside II	$C_{43}H_{70}O_{15}$	826.4715
58	astragaloside I	$C_{45}H_{72}O_{16}$	868.4820
59	isoastragaloside I	$C_{45}H_{72}O_{16}$	868.4820
Triterpenoids			
60	betulinol	$C_{30}H_{50}O_{2}$	442.3811
61	ursolic acid	$C_{30}H_{48}O_{3}$	456.3607
Phenolic acids			
62	salvianolic acid A	$C_{26}H_{22}O_{10}$	494.1213
63	dimethyl lithospermate B	$C_{38}H_{34}O_{16}$	746.1847
64	9'-methyl lithospermate B	$C_{37}H_{32}O_{16}$	732.1690
65	salvianolic acid B	$C_{36}H_{30}O_{16}$	718.1534
66	salvianolic acid F	$C_{17}H_{14}O_{6}$	314.0790
67	danshensu	$C_{9}H_{10}O_{5}$	198.0528
68	rosmarinic acid	$C_{18}H_{16}O_{8}$	360.0845
69	ferulic Acid	$C_{10}H_{10}O_{4}$	194.0579
Tanshitones			
70	tanshinone I	$C_{18}H_{12}O_{3}$	276.0786
71	cryptotanshinone	$C_{19}H_{20}O_{3}$	296.1412
72	tanshinone IIA	$C_{19}H_{18}O_{3}$	294.1256
Flavonoids			
73	buddleoside	$C_{28}H_{32}O_{14}$	592.1792
74	astragalin	$C_{21}H_{20}O_{10}$	432.1056

75	complannatuside	$C_{28}H_{32}O_{16}$	624.1690
76	isomucronulatol-7-O-b-D-glucoside	$C_{23}H_{28}O_{10}$	464.1682
77	formononetin glucoside	$C_{22}H_{22}O_{9}$	430.1264
78	formononetin	$C_{16}H_{12}O_{4}$	268.0736
79	calycosin	$C_{16}H_{12}O_{5}$	284.0685
80	calycosin-7-O-β-D-glucoside	$C_{22}H_{22}O_{10}$	446.1213
iridoids			
81	harpagoside	$C_{24}H_{30}O_{11}$	494.1788
82	harpagide	$C_{15}H_{24}O_{10}$	364.1369
83	aucubin	$C_{15}H_{22}O_{9}$	346.1264
Others			
84	protocatechualdehyde	$C_{7}H_{6}O_{3}$	138.0317
85	chrysophanol	$C_{15}H_{10}O_{4}$	254.0579

Table S2 Information of the 230 components characterized from CXC.

No.	Observed t_R (min)	Mass (*m/z*)	Formula	Mass error (ppm)	Observed CCS (Å²)	ESI-MS²	Adducts	Identification	NRR	AR	SMRR	SR
1[a]	1.62	391.1239	$C_{15}H_{22}O_9$	2.0	176.01	345.1195,183.0776, 166.0607,145.8899, 97.0370	+HCOO,-H	aucubin				√
2[a]	1.87	409.1348	$C_{15}H_{24}O_{10}$	-0.8	188.40	208.0763,183.0699, 165.0580,139.0410, 123.0470,97.0280	+HCOO,-H,+Cl	harpagide				√
3[a]	2.03	197.0449	$C_9H_{10}O_5$	-3.5	141.11	135.0449,123.0458, 109.0313	-H	danshensu			√	
4	2.06	179.0344	$C_8H_8O_4$	-3.1	139.47	135.0459	-H	3,4-dihydroxycinnamic acid or its isomer				√
5	2.34	487.1459	$C_{21}H_{28}O_{13}$	0.4	208.22	318.1190,179.0358, 135.0458,133.0327, 110.0275	-H	cistanoside F or its isomer				√
6	2.51	503.1409	$C_{21}H_{28}O_{14}$	-0.5	204.64	221.0815,179.0348, 161.0264	-H	6-O-caffeoyl-β-D-fructofuranosyl-2-α-D-glucopyranoside or its isomer				√
7	2.64	655.4416	$C_{36}H_{62}O_{10}$	0.1	266.71	637.4338,439.3586, 421.3479,143.1075		notoginsenoside ST7 or its isomer	√			
8	2.71	879.4967	$C_{42}H_{74}O_{16}$	1.0	303.36	833.4841,671.4450, 509.3907,391.2869,	+HCOO	vinaginsenoside R21 or its isomer	√			

						170.0984,113.0258					
9	2.77	607.2254	$C_{26}H_{40}O_{16}$	1.7	222.88	443.1670,283.0250, 256.1344,219.0735	-H	deferuloylangoroside C or its isomer			√
10	2.82	487.1458	$C_{21}H_{28}O_{13}$	0.2	215.32	307.0864,265.0692, 205.0502,163.0425, 145.0301 ,135.0446	-H	6-O-p-coumaroylsucrose or its isomer			√
11[a]	2.87	137.0241	$C_7H_6O_3$	-2.5	118.10	108.0205,93.0345,92.0265	-H	protocatechualdehyde or its isomer		√	
12	2.99	517.1569	$C_{22}H_{30}O_{14}$	1.1	221.38	193.0613,175.0418, 164.0786,132.0218	-H	6'-O-feruloylsucrose or its isomer			√
13	3.17	179.0352	$C_9H_8O_4$	1.3	—	135.0457,134.0369	-H,+CH_3COO	caffeic acid or its isomer		√	
14	3.20	861.4876	$C_{42}H_{72}O_{15}$	2.7	300.86	653.4252,635.4195, 491.3799,329.2547, 113.0255	+HCOO	OH-PPT+2Glc	√		
15	3.23	639.4460	$C_{36}H_{62}O_9$	-1.0	263,49	621.4391,533.3537, 423.3618,405.3623, 217.1967	+H,+Na	ginsenoside Rh13 or its isomer	√		
16	3.26	863.5017	$C_{42}H_{74}O_{15}$	0.8	305.56	493.3911,417.3457, 399.3331,323.2650, 113.0214	+HCOO	quinquenoside L9 or its isomer	√		
17	3.40	465.1025	$C_{21}H_{20}O_{12}$	-0.6	233.58	303.0490,257.0438	+H	hyperoside or its isomer	√		√
18	3.42	877.4822	$C_{42}H_{72}O_{16}$	2.2	305.57	553.3402,471.3509, 391.2907	+HCOO	floralginsenoside A or its isomer	√		
19	3.48	287.0545	$C_{15}H_{10}O_6$	-1.7	—	230.1615,213.0550,	+H	kaempferol or its	√		

						178.0626,153.0184		isomer			
20	3.64	859.4722	$C_{42}H_{70}O_{15}$	3.0	302.66	633.4673,593.3822, 471.3484,321.0469, 269.0952	+HCOO	vinaginsenoside R25 or its isomer	√		
21[a]	3.76	491.1201	$C_{22}H_{22}O_{10}$	1.3	230.68	283.0609,268.0369, 211.0411	+HCOO	calycosin-7-O-β-D-glucoside		√	
22	3.80	447.1281	$C_{22}H_{22}O_{10}$	-1.1	213.68	285.0746,270.0513, 253.0489,175.0399, 137.0229,109.0268	+H	campanulin or its isomer		√	
23	3.82	285.0746	$C_{16}H_{12}O_{5}$	-3.9	213.67	270.0514,253.0495, 213.0544,186.0696, 137.0231	+H	calycosin or its isomer		√	
24	3.92	623.1992	$C_{29}H_{36}O_{15}$	1.7	225.41	461.1718,443.1522, 315.1117,297.1003, 255.0305,161.0238, 133.0290	-H	acteoside or its isomer			√
25	4.00	509.1671	$C_{23}H_{28}O_{10}$	1.2	215.91	463.0892,187.0391, 161.0592,145.0312, 133.0705	+HCOO	2'-hydroxy-3',4'-dimenthoxy-isoflavan-7-O-β-glucoside or its isomer			√
26	4.11	769.2578	$C_{35}H_{46}O_{19}$	2.3	255.33	447.1582,429.1395, 315.1040,175.0412, 160.0231,135.0448	-H	scrophuloside B1 or its isomer			√
27	4.22	517.1567	$C_{21}H_{28}O_{12}$	0.7	214.59	245.0808,161.0622, 147.0456,131.0525, 103.0543	+HCOO,-H	sibirioside A			√
28	4.29	1139.5890	$C_{53}H_{90}O_{23}$	3.2	334.87	1093.5835,961.5403	+HCOO,-H	PPT+3Glc+Xyl	√		

						,637.4344,475.3800, 391.2890,161.0460					
29	4.44	623.1988	$C_{29}H_{36}O_{15}$	1.0	224.68	461.1681,315.1089, 161.0239	-H	verbascoside or its isomer			√
30	4.54	1139.5884	$C_{54}H_{92}O_{25}$	2.6	337.96	1094.5794,961.5398 ,769.4805,637.4474, 475.3882,391.2874, 221.0676	+HCOO,-H	PPT+(Glc-Glc)-Glc-Xyl	√		
31	4.73	1007.5458	$C_{48}H_{82}O_{19}$	2.5	318.74	961.5427,799.4873, 637.4356,553.3402, 475.3799,391.2861, 221.0683	+HCOO,+Cl	PPT+3Glc	√		
32	4.76	1139.5886	$C_{53}H_{90}O_{23}$	2.7	346.47	961.5362,799.4849, 637.4432,475.3838	+HCOO	PPT+3Glc+Xyl	√		
33	4.84	1153.6026	$C_{54}H_{92}O_{23}$	1.2	338.94	1107.5979,945.5502 ,783.4883,637.4333, 475.3814,391.2839, 161.0485	+HCOO	PPT+3Glc+Rha	√		
34	4.94	339.0509	$C_{18}H_{12}O_7$	-0.3	216.86	295.0630	-H	phenolic acids G		√	
35	4.96	537.1048	$C_{27}H_{22}O_{12}$	1.8	216.43	295.0624,185.0252. 159.0478,135.0478, 109.0312	-H	lithospermic acid or its isomer		√	
36[a]	5.01	961.5417	$C_{48}H_{82}O_{19}$	-4.1	316.36	781.4806,637.4371, 475.3801,391.2945, 221.0669	+HCOO	notoginsenoside N	√		
37	5.03	317.0662	$C_{16}H_{12}O_7$	1.8	—	302.0434,257.0487,	+H	isorhamnetin or its	√		

						201.0521,153.0198, 107.0480		isomer				
38	5.16	463.1246	$C_{22}H_{22}O_{11}$	2.4	—	301.0705,286.0510, 153.0243	+H	kaempferide-4'-methyl ether-3-Glc or its isomer		√		
39	5.17	283.0606	$C_{16}H_{12}O_{5}$	-2.1	166.53	268.0376,239.0355, 148.0178,135.0100, 92.0232	-H	calycosin or its isomer		√		
40	5.26	783.2737	$C_{36}H_{48}O_{19}$	2.5	267.23	607.2227,443.1569, 193.0518,175.0396, 160.0163,89.0293	-H	angoroside C or its isomer				√
41	5.31	717.1480	$C_{36}H_{30}O_{16}$	2.7	236.04	339.0473,321.0419, 295.0643,109.0313	-H	lithospermic acid B or its isomer			√	
42	5.36	1093.5780	$C_{53}H_{90}O_{23}$	1.8	338.38	1093.5780,638.4604 ,475.3826,391.3011, 245.0645	+HCOO,-H	PPT+3Glc+Xyl	√			
43[a]	5.37	1007.5452	$C_{48}H_{82}O_{18}$	1.9	326.94	961.5422,799.4872, 637.4335,391.2868, 221.0671,113.0249	+HCOO	20-*O*-glucosylginsenoside Rf	√			
44	5.43	783.2741	$C_{36}H_{48}O_{19}$	3.1	266.15	443.1562,193.0519, 175.0401,160.0167, 149.0602	-H	angoroside C or its isomer				√
45	5.44	717.1471	$C_{36}H_{30}O_{16}$	1.4	240.58	339.0527,321.0397, 295.0613,185.0251	-H	lithospermic acid B isomer			√	
46	5.49	1007.5446	$C_{48}H_{82}O_{19}$	1.4	326.94	962.5590,799.5172, 637.3781,475.3781,	+HCOO	PPT+3Glc	√			

						205.9048				
47	5.50	831.4756	$C_{41}H_{70}O_{14}$	1.2	289.49	635.4296,491.3742, 391.2851	+HCOO	OH-PPT+Glc+Xyl	√	
48	5.50	843.4755	$C_{42}H_{70}O_{14}$	0.9	298.84	554.3435,391.2848	+HCOO	dehydro-PPT-2Glc	√	
49[a]	5.74	977.5334	$C_{47}H_{80}O_{18}$	0.8	246.13/317.59	799.4900,637.4317, 475.3785,391.2845	-H,+HCOO	notoginsenoside R1	√	
50	5.83	831.4769	$C_{41}H_{70}O_{14}$	2.7	—	653.4336,491.3761, 391.2859,389.2714	+HCOO	OH-PPT-Glc-Xyl	√	
51	5.83	1007.5457	$C_{48}H_{82}O_{19}$	2.6	317.59	961.5947,587.0089, 475.3793,391.3000	+HCOO	PPT+3Glc	√	
52	5.86	977.5345	$C_{48}H_{82}O_{20}$	1.9	327.48	932.5301,799.4863, 637.4350,475.3787, 391.2846,101.0269	-H,+HCOO	PPT+2Glc+Xyl	√	
53	5.88	699.4333	$C_{36}H_{62}O_{10}$	1.1	263.64	637.4309,475.3787, 391.2811	+HCOO	OH-PPT+Glc	√	
54	5.91	493.1146	$C_{26}H_{22}O_{10}$	1.1	209.35	295.0612,185.03401 59.0453,135.0449,1 09.0291	-H	salvianolic acid A isomer		√
55	5.97	537.1060	$C_{27}H_{22}O_{12}$	4.0	—	295.0631,185.0239. 159.0451,135.0439, 109.0294	-H	lithospermic acid or its isomer		√
56	5.98	977.5352	$C_{47}H_{80}O_{18}$	2.7	322.18	931.5271,769.4772, 637.4350,475.3803, 391.2856	+HCOO,-H	PPT+2Glc+Xyl	√	
57	6.01	1167.5842	$C_{26}H_{22}O_{10}$	1.1	348.63	1121.5873,959.5363 ,797.4683,589.4183,	+HCOO,-H	dehydro-PPT-4Glc	√	

						473.3710,221.0683				
58	6.07	991.5508	$C_{48}H_{82}O_{18}$	2.5	328.83	946.5496,783.4970, 637.4307,475.3821, 391.2861,311.1030	+HCOO	PPT+2Glc+Rha	√	
59	6.10	1007.5461	$C_{48}H_{82}O_{19}$	2.9	321.36	961.5412,637.4385, 475.3832,323.1034	+HCOO,-H	PPT+3Glc	√	
60	6.12	1301.6436	$C_{59}H_{100}O_{28}$	4.1	374.16	1255.6414,1144.5049,961.5493,799.4990,637.4511,475.3916,353.1110,221.0652	+HCOO	PPT+4Glc+Xyl	√	
61	6.16	977.5353	$C_{47}H_{80}O_{18}$	2.7	322.18	931.5297,769.4852, 637.4369,475.3816, 391.2858	+HCOO,-H	PPT+2Glc+Xyl	√	
62[a]	6.39	845.4916	$C_{42}H_{72}O_{14}$	1.4	297.33	637.4334,475.3784, 391.2852	+HCOO,+Cl	ginsenoside Rg1	√	
63[a]	6.43	991.5503	$C_{48}H_{82}O_{18}$	2.0	312.67	945.5578,783.4928, 637.4346,475.3790, 391.2883	+HCOO	ginsenoside Re	√	
64[a]	6.72	717.1469	$C_{36}H_{30}O_{16}$	1.1	246.53	519.0918,339.0504, 321.0394,295.0560, 185.0236	-H	salvianolic acid B		√
65	7.12	845.4922	$C_{42}H_{72}O_{14}$	2.1	298.47	637.4400,475.3806, 391.2871	+HCOO	PPT+2Glc	√	
66	7.13	717.1474	$C_{36}H_{30}O_{16}$	0.6	246.02	321.0403,295.0620, 277.0517,185.0251,	-H	salvianolic acid B isomer		√

						109.0303					
67[a]	7.19	841.4981	$C_{44}H_{74}O_{15}$	3.2	297.48	637.4297,619.4216, 554.3469,475.3840, 391.2831	+HCOO,-H	notoginsenoside Rt	√		
68	7.28	1031.5457	$C_{51}H_{84}O_{21}$	2.4	325.23	783.4914,637.4408, 475.3857,351.2960	-H	PPT+2Glc+Rha+Mal.	√		
69	7.31	717.1477	$C_{36}H_{30}O_{16}$	2.2	241.91	339.0511,321.0402, 279.0311,185.0253, 135.0472,109.0310	-H	salvianolic acid B isomer			√
70	7.34	843.4776	$C_{42}H_{70}O_{14}$	3.4	299.89	637.4311,473.3675, 389.2703,229.0430	+HCOO	dehydro-PPT-2Glc	√		
71	7.41	507.1509	$C_{23}H_{26}O_{10}$	0.3	234.72	321.0417,284.0694, 269.0476,241.0495, 145.0324	+HCOO	9,10-dimethoxy-pterocapan-3-O-β-glucoside or its isomer		√	
72	7.64	1005.5302	$C_{48}H_{80}O_{19}$	2.6	335.97	959.5242,797.4718, 635.4146,473.3637, 389.2708	+HCOO	dehydro-PPT-3Glc	√		
73[a]	7.70	493.1144	$C_{26}H_{22}O_{10}$	0.7	208.49	295.0594,267.0712, 185.0257,159.0452, 109.0309	-H	salvianolic acid A			√
74	7.84	463.1614	$C_{23}H_{28}O_{10}$	0.9	218.07	301.1109,286.0847, 254.0545,149.0241	-H	partensein-7-O-Glc-6"-O-malonate or its isomer		√	
75[a]	7.86	815.4820	$C_{41}H_{70}O_{13}$	1.8	294.58	607.4214,475.3838, 391.2866	+HCOO	pseudoginsenoside Rt3	√		
76	7.88	887.5026	$C_{44}H_{74}O_{15}$	1.9	310.25	637.4403,619.4175,	+HCOO,-H	PPT+2Glc+Ac	√		

						475.3874,391.2886, 329.2886						
77[a]	7.98	539.1777	$C_{24}H_{30}O_{11}$	1.2	226.14	493.1719,345.1213, 165.0553,147.0446, 121.0299,103.0548	+HCOO,-H	harpagoside				√
78	8.14	1167.5840	$C_{54}H_{90}O_{24}$	3.1	358.43	1121.5723,959.5312 ,867.4843,797.4801, 635.4323,473.3659, 389.2742,221.0685, 161.0449	+HCOO,-H	dehydro-PPT-4Glc	√			
79	8.19	551.1210	$C_{28}H_{24}O_{12}$	2.7	219.40	321.0380,303.0258, 266.0643	-H	monomethyl lithospermate or its isomer			√	
80[a]	8.27	283.0604	$C_{16}H_{12}O_{5}$	-2.9	166.81	268.0368,239.0349, 195.0457,148.0162, 135.0114,91.0206.	-H	calycosin		√		
81	8.28	961.5390	$C_{47}H_{80}O_{17}$	1.4	325.84	915.5310,783.4958, 637.4328,619.4254, 475.3815,391.3815, 161.0446	+HCOO,-H	PPT+Glc+Rha+Xyl	√			
82	8.34	887.5035	$C_{44}H_{74}O_{15}$	2.9	—	842.5045,620.4384, 544.8985	+HCOO,-H	PPT+2Glc+Ac	√			
83[a]	8.34	815.4818	$C_{41}H_{70}O_{13}$	2.4	297.26	637.4330,553.3413, 475.3823,391.2883, 264.0925	+HCOO	sanchinoside A3	√			
84[a]	8.45	815.4819	$C_{41}H_{70}O_{13}$	2.5	305.88	637.4352,553.3422,	+HCOO	6-O-(β-D-glucopyrano	√			

						475.3824,391.2836		syl)-20-O-(β-D-Xylopyranosyl)-3β,6α,12β,20(S)-tetrahydroxy dammar-24-ene		
85	8.53	887.5032	$C_{44}H_{74}O_{15}$	2.6	305.89	637.4406,619.4269, 553.3395,475.3822, 391.2875	+HCOO,+Cl	PPT+2Glc+Ac	√	
86	8.60	1007.5459	$C_{48}H_{82}O_{19}$	2.8	336.34	961.5305,799.4923, 781.4773,619.4202, 537.3491,383.1244, 221.0712	+HCOO	PPT+3Glc	√	
87	8.83	843.4766	$C_{42}H_{70}O_{14}$	2.2	300.40	635.4241,475.3795, 345.2451	+HCOO	dehydro-PPT-2Glc	√	
88[a]	8.85	1007.5457	$C_{48}H_{82}O_{18}$	2.5	335.36	961.5408,799.4875, 637.4343,475.3802, 391.2875	+HCOO	vinaginsenoside R4	√	
89	8.87	491.0986	$C_{26}H_{20}O_{10}$	0.4	202.72	311.0564,294.0554, 135.0465	-H	salvianolic acid C or its isomer		√
90	8.95	977.5347	$C_{47}H_{80}O_{18}$	2.1	324.22	931.5130,475.3798, 353.1097,221.0690	+HCOO,-H	PPT+(Glc-Glc)+Xyl	√	
91	9.18	565.1358	$C_{28}H_{24}O_{10}$	1.1	223.46	339.0546,321.0402, 293.0453,277.0507, 185.0253,135.0459, 109.0297	+HCOO	lithospermic acid		√
92	9.30	827.4821	$C_{42}H_{70}O_{13}$	2.7	310.69	457.3768,403.8583	+HCOO	dedihydro-PPT+2Glc	√	
93	9.40	505.1726	$C_{25}H_{30}O_{11}$	2.1	225.58	301.1065,286.0872,	-H,+HCOO	Rhamnocitin-Hex-acet		√

						271.0573,135.0445,121.0294		ate or its isomer	
94	9.45	887.5026	$C_{44}H_{74}O_{15}$	1.9	312.14	637.4401,475.3811,391.2925,377.2794	+HCOO	PPT+2Glc+Ac	√
95	9.53	829.4965	$C_{42}H_{72}O_{13}$	1.2	301.41	637.4348,553.3377,475.3930	+HCOO	PPT+Glc+Rha	√
96[a]	9.56	1417.6899	$C_{64}H_{108}O_{31}$	3.0	399.72	1371.6851,1239.6423,1107.6004,945.5476,783.4930,621.4387,459.3878,353.1088,221.0664,161.0470	+HCOO,-H	notoginsenoside T	√
97	9.71	845.4921	$C_{42}H_{72}O_{14}$	1.9	295.27	799.4922,621.4505,475.3859,391.2753	+HCOO	PPT+2Glc	√
98	9.79	1315.6578	$C_{60}H_{102}O_{28}$	2.9	372.75	1269.6577,1107.6041,945.5547,783.5075,621.4410,544.5564,383.1179,221.0681	+HCOO	PPD+5Glc	√
99	9.82	831.4787	$C_{41}H_{70}O_{14}$	0.6	299.58	785.4723,491.3752,415.3202,161.0446	+HCOO	OT+Glc+Xyl	√
100	9.90	1315.6579	$C_{60}H_{102}O_{28}$	3.0	370.26	1269.6521,1107.6032,945.5502,783.4958,621.4452,459.3849,383.1203,221.0681	+HCOO,-H	PPD+5Glc	√

101[a]	10.01	845.4924	$C_{42}H_{72}O_{14}$	2.4	307.64	799.4849,637.4358, 475.3797,391.2859, 101.0239	+HCOO,-H	ginsenoside Rf	√
102	10.11	1005.5303	$C_{48}H_{80}O_{19}$	2.7	335.07	959.5297,797.4717, 635.4335,473.3679, 449.3369,101.0224	+HCOO	dehydro-PPT+3Glc	√
103	10.13	845.4923	$C_{42}H_{72}O_{14}$	2.2	301.00	799.4793,783.5052, 475.3809,391.2956	+HCOO	PPT+2Glc	√
104[a]	10.21	1285.6477	$C_{59}H_{100}O_{27}$	3.4	372.70	1239.6426,1107.5989,783.4929,621.4382,459.3853,353.1084,221.0660,161.0452	+HCOO	notoginsenoside R4	√
105	10.30	845.4919	$C_{42}H_{72}O_{14}$	1.8	301.00	799.4921,637.4346, 475.3802,391.2876	+HCOO,+Cl	PPT+2Glc	√
106	10.41	1315.6579	$C_{60}H_{102}O_{28}$	3.0	381.19	1269.6589,1107.5975,945.5619,783.4866,621.4537,460.3869,221.0679,161.0448	+HCOO	PPD+5Glc	√
107	10.53	1315.6564	$C_{60}H_{102}O_{28}$	1.9	372.90	1269.6540,1107.6023,945.5552,783.4958,459.3885,383.1228,221.0683	+HCOO,-H	PPD+5Glc	√
108	10.59	605.4416	$C_{36}H_{60}O_{7}$	0.7	263.00	587.4369,407.3639, 189.1636	+H	ginsenoside Pk2 or its isomer	√

109	10.62	947.5237	$C_{46}H_{78}O_{17}$	1.7	323.34	769.4698,475.3824,<br>323.0996,191.0561,<br>131.0350	+HCOO	PPT+Glc+2Xyl	√
110	10.65	1417.6894	$C_{64}H_{108}O_{31}$	2.7	389.76	1371.6829,1107.599<br>3,945.5485,783.492<br>7,459.3829,621.443<br>5,353.1136,221.066<br>5	+HCOO,-H	PPD+4Glc+2Xyl	√
111[a]	10.65	1285.6466	$C_{59}H_{100}O_{28}$	2.5	373.87	1239.6430,1107.599<br>0,945.5453,783.492<br>5,621.4385,459.383<br>2,221.0679,161.045<br>8	+HCOO,-H	notoginsenoside Fa	√
112	10.71	977.5362	$C_{47}H_{80}O_{18}$	3.7	326.47	799.4964,637.4393,<br>475.3844,355.2609	+HCOO,-H	PPT+2Glc+Xyl	√
113[a]	10.77	815.4807	$C_{41}H_{70}O_{13}$	0.8	300.00	637.4331,475.37833<br>,391.2849	+HCOO,-H	notoginsenoside R2	√
114[a]	10.96	1151.5870	$C_{54}H_{90}O_{23}$	1.3	352.03	1106.5861,943.5339<br>,781.4694,763.4690,<br>457.3883,373.2840,<br>221.0746	+HCOO	5,6-didehydroginsenosi<br>de Rb1	√
115[a]	11.36	815.4821	$C_{41}H_{70}O_{13}$	1.6	305.39	621.4634,553.3415,<br>475.3795,391.2877	+HCOO	20(*R*)-notoginsenoside<br>R2	√
116	11.45	767.4943	$C_{42}H_{70}O_{12}$	0.4	294.91	533.4025,423.3640,<br>187.1493	+H	ginsenoside Pk1 or its<br>isomer	√
117[a]	11.34	1285.6465	$C_{59}H_{100}O_{27}$	2.4	368.36	1239.6441,1107.599	+HCOO,-H	ginsenoside Ra3	√

						0,945.5373,783.4948,621.4416,459.3870,221.0671			
118[a]	11.50	1153.6043	$C_{54}H_{92}O_{23}$	2.7	359.08	1107.5966,945.5447,783.4910,621.4378,459.3850,161.0457	+HCOO,-H	ginsenoside Rb1	√
119[a]	11.52	829.4970	$C_{42}H_{72}O_{13}$	1.9	301.09	783.4906,637.4344,475.3792,391.2853,161.0479	+HCOO	ginsenoside Rg2	√
120[a]	11.83	683.4378	$C_{36}H_{62}O_{9}$	0.2	276.80	554.3367,475.3789,391.2903,350.2836	+HCOO,+Cl	ginsenoside Rh1	√
121	11.92	1285.6448	$C_{59}H_{100}O_{28}$	1.1	—	1239.6374,1107.5967,945.5449,783.4887,621.4386,459.3829	+HCOO,-H	PPD+4Glc+Xyl	√
122[a]	11.97	1193.5979	$C_{57}H_{94}O_{26}$	1.5	356.57	1149.6198,1107.5975,945.5432,783.4921,621.4369,459.3857,221.0744	-H	malonyl-ginsenoside Rb1	√
123[a]	12.01	1255.6347	$C_{58}H_{98}O_{26}$	1.5	359.20	1209.6255,1077.5910,945.5577,783.4837,621.4400,459.3837,323.1037,131.0366	+HCOO	ginsenoside Ra1	√
124	12.02	1153.6029	$C_{54}H_{92}O_{23}$	1.5	350.27	1107.6029,945.5581,783.4938,621.4457,	+HCOO	PPD+4Glc	√

						459.3904,375.2901			
125	12.07	1193.5983	$C_{57}H_{94}O_{26}$	1.9	348.12	1108.6050,946.5570,784.4977,621.4442,221.0745	-H	PPD+4Glc+Mal.	√
126	12.08	621.4357	$C_{36}H_{60}O_{8}$	-0.6	263.42	604.4383,509.3149,405.3505,363.3061,269.2268	+H	dedihydro-PPT+Glc	√
127[a]	12.09	1123.5940	$C_{53}H_{90}O_{22}$	3.1	347.77	1107.6030,946.5398,783.4839,621.4414,459.3876,221.0766	+HCOO	ginsenoside Rc	√
128	12.26	1087.5354	$C_{53}H_{84}O_{23}$	2.1	364.54	955.5290,731.4382,569.3859,551.3740,455.3532	-H	OA+GlcA+2Glc+Xyl	√
129	12.26	1193.5992	$C_{57}H_{94}O_{26}$	2.6	351.90	1107.6053,945.5376,783.5041,621.4439,459.3808,,221.0683	-H	PPD+4Glc+Mal.	√
130[a]	12.27	683.4378	$C_{36}H_{62}O_{9}$	0.3	282.09	637.4532,475.3834,391.2861	+HCOO	20(*R*)-ginsenoside Rh1	√
131	12.32	857.4917	$C_{43}H_{72}O_{14}$	1.6	310.27	770.4897,637.4270,475.3802,391.2771	+HCOO,-H,+CH_3COO	notoginsenoside SP17 or its isomer	√
132[a]	12.37	955.4923	$C_{48}H_{76}O_{19}$	1.6	325.99	793.4412,613.3781,569.3848,523.3813,455.3568,397.1445	-H	ginsenoside Ro	√
133	12.40	871.4069	$C_{44}H_{74}O_{14}$	1.0	311.30	783.5000,475.3813,391.2880	+HCOO	PPT+Glc+Rha+Ac	√
134	12.45	1195.6138	$C_{56}H_{94}O_{24}$	1.7	372.56	1149.6436,1107.597	+HCOO	PPD+4Glc+Ac	√

						7,925.4827,783.5041,621.4396,459.3868,221.0720,161.0457				
135	12.46	1087.5350	$C_{53}H_{84}O_{23}$	1.8	213.91/367.01	925.4810,731.4412,551.3746,455.3526,453.3383	-H	OA+GlcA+2Glc+Xyl	√	
136[a]	12.64	1123.5925	$C_{53}H_{90}O_{22}$	1.8	362.99	1107.5926,945.5545,783.4876,621.4422,459.3891,375.2929	+HCOO	ginsenoside Rb2	√	
137	12.64	1279.5987	$C_{60}H_{95}O_{29}$	1.8	364.31	1136.5433,1107.5926,945.5545,783.4876,621.4422,459.3891,375.2929	+HCOO	PPD+4Xyl+Glc+2Ac.	√	
138	12.69	1193.5999	$C_{57}H_{94}O_{26}$	3.2	365.31	1107.6026,945.5461,783.4905,621.4405,459.3890	-H	PPD+4Glc+Mal.	√	
139[a]	12.75	1123.5932	$C_{53}H_{90}O_{22}$	2.4	349.65	1077.5861,945.5493,783.4925,621.4386,459.3904,191.0587	+HCOO	ginsenoside Rb3	√	
140	12.85	955.4940	$C_{48}H_{76}O_{19}$	-2.8	337.33	793.4486,621.4534,569.3909,455.3516	-H	OA+GlcA+2Glc	√	
141	12.85	725.4499	$C_{38}H_{64}O_{10}$	2.6	285.84	475.3845,391.2931	+HCOO	PPT+Glc+Ac	√	
142[a]	12.90	829.4612	$C_{41}H_{68}O_{14}$	2.5	303.52	475.3833,331.2360	+HCOO	astragaloside IV		√
143	13.02	1193.5991	$C_{57}H_{94}O_{26}$	2.6	362.91	1107.5987,945.5458,783.4928,621.4417,	-H	PPD+4Glc+Mal.	√	

						459.3876,221.0710				
144	13.09	1123.5922	$C_{53}H_{90}O_{22}$	1.4	362.20	1078.5862,945.5444,783.4904,621.4384,459.3850,375.2873,161.0482	+HCOO	PPD+3Glc+Xyl	√	
145	13.16	1153.6036	$C_{54}H_{92}O_{23}$	2.2	363.75	1107.5992,945.5750,783.4928,621.4433	+HCOO	PPD+4Glc	√	
146[a]	13.18	829.4606	$C_{41}H_{68}O_{14}$	1.8	281.56	783.4495,621.4250,489.3590,383.2930,101.0249	+HCOO	astragaloside III		√
147[a]	13.23	267.0657	$C_{16}H_{12}O_{4}$	-2.3	162.40	253.0459,252.0421,223.0401,195.0454,132.0208	-H	formononetin		√
148	13.27	1123.5937	$C_{53}H_{90}O_{22}$	2.8	342.08	1077.5914,621.4478,475.3814,353.1099,221.0685	+HCOO	PPD+3Glc+Xyl	√	
149	13.29	785.4708	$C_{40}H_{68}O_{12}$	1.9	291.97	475.3794,391.2868	+HCOO,+CH_3COO	PPT+2Xyl	√	
150	13.30	989.5346	$C_{48}H_{80}O_{18}$	2.0	331.34	943.5269,781.4763,619.4230,457.3713,373.2768	+HCOO,-H	dedihydro-PPT+3Glc	√	
151	13.40	1195.6140	$C_{56}H_{94}O_{24}$	1.9	363.25	1107.5995,945.5453,783.4936,621.4382,459.3899,221.0669	+HCOO,-H	PPD+4Glc+Ac	√	
152[a]	13.59	683.4379	$C_{36}H_{62}O_{9}$	0.4	275.21	475.3812,391.2874,347.2605	+HCOO,+Cl	ginsenoside F1	√	

153	13.63	1195.6135	$C_{56}H_{94}O_{24}$	1.5	366.49	1107.6004,945.5447,783.4886,621.4349,459.3862	+HCOO,-H	PPD+4Glc+Ac	√
154	13.77	725.4484	$C_{38}H_{64}O_{10}$	0.4	286.38	475.3846	+HCOO	PPT+Glc+Ac	√
155[a]	14.06	991.5501	$C_{48}H_{82}O_{18}$	1.8	336.42	945.5449,783.4905,621.4382,459.3831,375.2834	+HCOO	ginsenoside Rd	√
156	14.23	975.5545	$C_{48}H_{82}O_{17}$	1.2	327.21	929.5479,605.4477,439.4477	+HCOO	dehydro-PPT+2Glc+Xyl	√
157	14.38	989.5332	$C_{48}H_{80}O_{18}$	0.6	322.11	943.5426,765.4794,621.4343,457.3658,161.0454	+HCOO	dedihydro-PPT+3Glc	√
158	14.39	991.5491	$C_{48}H_{82}O_{18}$	0.8	334.96	945.5462,783.4874,621.4395,459.3880,375.2948	+HCOO	PPD+3Glc	√
159	14.53	1031.5447	$C_{51}H_{84}O_{21}$	1.4	341.92	945.5447,783.4908,621.4380,459.3868,375.2932	-H	PPD+3Glc+Mal	√
160	14.59	793.4393	$C_{42}H_{66}O_{14}$	1.6	288.43	631.3899,569.3871,455.3553,389.2830	-H	OA+GlcA+Glc	√
161	14.71	1031.5443	$C_{51}H_{84}O_{21}$	1.0	324.78	945.5441,783.4917,621.4379,459.3860,375.2930	-H	PPD+3Glc+Mal	√
162	14.79	1033.5591	$C_{50}H_{84}O_{19}$	0.2	345.13	987.5441,783.4939,621.4395,459.3874,375.2929	+HCOO,-H	PPD+3Glc+Ac	√

163	14.86	725.4494	$C_{38}H_{64}O_{10}$	1.7	285.36	638.4404,475.3810, 391.2865	+HCOO	PPT+Glc+Ac	√	
164[a]	15.18	991.5501	$C_{48}H_{82}O_{18}$	1.8	323.82	783.4826,621.4423, 459.3872,221.0690	+HCOO	gypenoside XVII	√	
165[a]	15.26	871.4631	$C_{43}H_{70}O_{15}$	-2.3	311.74	621.4323,603.4466, 537.3485,496.9952	+HCOO	astragaloside II		√
166	15.33	1031.5458	$C_{51}H_{84}O_{21}$	2.5	—	945.5397,783.4941, 621.4490,459.3918, 375.2967	-H	PPD+3Glc+Mal	√	
167	15.38	991.5503	$C_{48}H_{82}O_{18}$	2.0	—	621.4512,459.3851, 376.2899,101.0262	+HCOO	PPD+3Glc	√	
168	15.47	1033.5604	$C_{50}H_{84}O_{19}$	1.5	342.84	987.5611,783.4942, 621.4407,459.3898, 375.2914	+HCOO	PPD+3Glc+Ac	√	
169	15.51	1031.5461	$C_{51}H_{84}O_{21}$	2.8	337.17	945.5451,783.4976, 621.4392,459.3889, 375.2938	-H	PPD+3Glc+Mal	√	
170[a]	15.54	725.4491	$C_{38}H_{64}O_{10}$	1.2	286.39	475.3869,391.2920	+HCOO	6-O-β-D-(6'-acetyl)-glucopyranosyl-24-en-dammar-3β,6α,12β,20S-tetraol	√	
171	15.75	961.5394	$C_{47}H_{80}O_{17}$	1.8	328.38	915.5360,621.4400, 537.3552,459.3876, 375.2915	+HCOO	PPD+2Glc+Xyl	√	
172	15.86	1031.5462	$C_{51}H_{84}O_{21}$	2.9	342.00	945.5533,783.4969, 621.4372,459.3855,	-H	PPD+3Glc+Mal	√	

						375.3000					
173	15.89	991.5507	$C_{48}H_{82}O_{18}$	2.4	333.96	945.5556,783.4943, 621.4434,357.2832	+HCOO	PPD+3Glc	√		
174	16.22	941.5132	$C_{48}H_{78}O_{18}$	1.9	344.64	941.5136,923.5072, 615.3940,457.3683, 205.0780	-H,+HCOO	soyasponin I isomer		√	
175[a]	16.29	961.5398	$C_{47}H_{80}O_{17}$	2.1	326.87	915.5355,783.4910, 621.4378,459.3850, 375.2901	-H,+HCOO	notoginsenoside Fe	√		
176	16.35	683.4387	$C_{36}H_{62}O_{9}$	1.7	274.11	475.3829,391.2886	+HCOO	PPT+Glc	√		
177	16.39	1031.5451	$C_{51}H_{84}O_{21}$	1.8	319.62	945.5464,765.4780, 621.4422,459.3845	-H	PPD+3Glc+Mal	√		
178	16.45	1033.5601	$C_{50}H_{84}O_{19}$	1.2	345.49	945.5427,783.4903, 621.4405,459.3906, 161.0484	+HCOO	PPD+3Glc+Ac	√		
179	16.47	961.5389	$C_{47}H_{80}O_{17}$	1.2	327.34	915.5356,783.4906, 621.4388,459.3861, 375.2983	+HCOO	PPD+2Glc+Xyl	√		
180	16.74	311.1271	$C_{19}H_{18}O_{4}$	-2.3	171.64	296.1066,283.1334, 265.1223,195.0444	+H	tanshinone II_B or its isomer			√
181[a]	16.95	797.4703	$C_{41}H_{68}O_{12}$	1.4	299.76	619.4245,457.3745	+HCOO,-H	notoginsenoside T5	√		
182	17.22	681.4239	$C_{36}H_{60}O_{9}$	2.9	—	473.3576,214.0439	+HCOO	dehydro-PPT+Glc	√		
183	17.39	811.4856	$C_{42}H_{70}O_{12}$	0.9	301.13	765.4817,457.3705, 257.0243,161.0486	+HCOO	dedihydro-PPT+Glc+Rha	√		
184	17.50	1033.5603	$C_{50}H_{84}O_{19}$	1.4	345.11	945.5457,783.4987, 621.4386,459.3861,	+HCOO	PPD+3Glc+Ac	√		

						373.2840					
185	17.50	797.4701	$C_{41}H_{68}O_{12}$	1.1	307.14	751.4662,619.4246, 457.3689	+HCOO,-H	dedihydro-PPT+Glc+Xyl	√		
186	17.74	293.1165	$C_{19}H_{16}O_{3}$	2.5	165.72	275.1080,247.1147, 219.1159		1,2-didehydrotanshinone II_A or its isomer			√
187	17.88	311.1273	$C_{19}H_{18}O_{4}$	-1.6	—	275.1069,252.1145, 247.2122,237.0924.	+H,+Na	hydroxytanshinone II_A or its isomer			√
188[a]	17.90	811.4861	$C_{42}H_{70}O_{12}$	1.5	308.09	765.4813,619.4247, 457.3669	+HCOO	ginsenoside F4	√		
189	18.17	975.5550	$C_{48}H_{82}O_{17}$	1.6	329.72	930.5518,621.4422, 459.3854,375.2937	+HCOO	PPD+2Glc+Rha	√		
190[a]	18.36	665.4275	$C_{36}H_{60}O_{8}$	0.8	276.22	619.4294,457.3813	+HCOO	ginsenoside Rk3	√		
191	18.46	827.4807	$C_{42}H_{70}O_{13}$	1.1	298.14	781.4775,620.4335, 439.3590,161.0511	+HCOO	dedihydro-PPT+2Glc	√		
192[a]	18.62	829.4963	$C_{42}H_{72}O_{13}$	1.1	307.68	783.4894,621.4365, 459.3870,375.2860	+HCOO	ginsenoside F2	√		
193[a]	18.87	665.4281	$C_{36}H_{60}O_{8}$	1.7	281.91/343.89	619.4262,458.3711	+HCOO	ginsenoside Rh4	√		
194[a]	18.89	961.5419	$C_{47}H_{80}O_{17}$	1.9	329.59	783.4935,621.4379, 459.3897,375.2970	+HCOO	notoginsenoside Ft1	√		
195	18.99	925.4823	$C_{47}H_{74}O_{18}$	2.2	339.23	745.4179,551.3765, 455.3541	-H	OA+GlcA+glx+Xyl	√		
196	19.02	311.2223	$C_{18}H_{32}O_{4}$	1.6	180.14	309.2089,293.2120, 223.1703.179.0004.	-H	dihydroxyoctadecadienoic acid or its isomer		√	
197[a]	19.19	891.4720	$C_{45}H_{72}O_{16}$	0.8	315.26	713.4242,447.0689, 299.5464	+Na	astragaloside I		√	

198	19.38	925.4812	$C_{47}H_{74}O_{18}$	1.1	343.55	731.4389,551.3757, 455.3543	-H	OA+GlcA+glx+Xyl	√			
199	19.44	295.0970	$C_{18}H_{16}O_4$	-1.9	169.41	249.0944,237.0929, 222.0676	-H	danshenxinkun A or its isomer			√	
200	19.67	325.1081	$C_{18}H_{16}O_3$	-0.2	—	265.0879,237.0923, 223.0794,165.0009, 152.9919	-H	methylendihydrotan-shinquinone or its isomer			√	
201	19.67	341.1379	$C_{20}H_{20}O_5$	1.3	179.63	295.1387,263.1069, 235.1112,207.1171	+H	trijuganone C or its isomer			√	
202	19.86	829.4967	$C_{42}H_{72}O_{13}$	1.5	305.93	783.4910,621.4374, 459.3834,375.2901	+HCOO	20(*S*)-ginsenoside Rg3	√			
203	19.95	825.4649	$C_{43}H_{70}O_{15}$	0.9	317.92	779.4676,617.4093, 455.3519,221.0669	-H,+HCOO,+CH3COO	OA+2Glc	√			
204	20.04	829.4969	$C_{42}H_{72}O_{13}$	1.7	302.45	783.4922,621.4391, 459.3857,375.2925	+HCOO,-H	PPD+2Glc	√			
205[a]	20.15	829.4969	$C_{42}H_{72}O_{13}$	1.7	302.08	783.4912,621.4395, 537.3442,459.3837, 375.2911	+HCOO,-H	20(*R*)-ginsenoside Rg3	√			
206[a]	20.18	913.4814	$C_{45}H_{72}O_{16}$	1.3	327.79	783.4900,621.4417, 434.0205	+HCOO,-H	isoastragaloside I		√		
207	20.38	811.4864	$C_{42}H_{70}O_{12}$	1.9	318.77	765.4808,603.4268, 456.9641	+HCOO,-H	dedihydro-PPT+Glc+Rha	√			
208	20.75	871.5085	$C_{44}H_{74}O_{14}$	3.0	—	621.4293,459.3843, 375.2843	+HCOO,-H	PPD+Glc+Glc+Ac	√			
209	20.93	885.2837	$C_{42}H_{48}O_{18}$	1.7	321.80	563.1931,461.1478, 279.2341,177.0548.	+HCOO	scrophuloside B4 or its isomer				√

210	21.60	943.5285	$C_{48}H_{80}O_{18}$	1.4	—	765.4840,603.4338		quinquen ide L1 or its isomer	√		
211	22.25	279.1006	$C_{18}H_{14}O_{3}$	-3.6	—	579.1780,261.0916, 233.0953,205.1005	+H,+Na	dihydrotanshinone I or its isomer			√
212	22.38	279.1019	$C_{18}H_{16}O_{3}$	-2.8	—	264.0814,251.1079, 222.0694,204.9884	-H	methylenedihydrotan-shinquinone or its isomer			√
213	22.50	281.1163	$C_{18}H_{16}O_{3}$	-3.3	—	263.1074,235.1109	+H,+Na	trijuganone B or its isomer			√
214[a]	22.73	811.4874	$C_{42}H_{70}O_{12}$	3.2	317.08	765.4824,603.4341, 439.3624,113.0248	+HCOO,-H	ginsenoside Rk1	√		
215	22.93	339.1218	$C_{20}H_{18}O_{5}$	-2.6	—	261.0899,233.0951, 205.1006,190.0774	+H,+Na	methyl tanshinonate or its isomer			√
216[a]	22.96	811.4863	$C_{42}H_{70}O_{12}$	1.8	324.71	765.4819,603.4284, 441.3728	+HCOO,-H	ginsenoside Rg5	√		
217[a]	23.39	667.4434	$C_{36}H_{62}O_{8}$	1.1	275.82	636.1689,414.9775	+HCOO,-H	compound K	√		
218	23.57	277.0849	$C_{18}H_{12}O_{3}$	3.7	—	249.0901,234.0668, 193.1004,178.0767	+H,+Na	tanshinone I isomer			√
219	23.89	311.1272	$C_{19}H_{18}O_{4}$	1.9	173.42	283.1352,267.1388, 251.1076,197.0606, 141.0749	+H,+Na	tanshinone II_B or its isomer			√
220[a]	23.89	667.4444	$C_{36}H_{62}O_{8}$	2.6	292.54	543.0358,395.9846.	+HCOO,-H	20(*R*)-ginsenoside Rh2	√		
221[a]	24.14	277.0849	$C_{18}H_{12}O_{3}$	-3.6	152.32	277,0882,249.0904, 234,0670,178.0770, 169.0662,141.0717	+H,+Na	tanshinone I			√
222	24.12	295.2275	$C_{18}H_{32}O_{3}$	1.2	181.03	277.2172,269.1532,	-H	13-hydroxy-9,11-octad		√	

						227.1072,195.1399, 171.1032		ecadienoic acid or its isomer		
223[a]	24.21	297.1477	$C_{19}H_{12}O_3$	-2.8	168.90	279.1369,267.1008, 251.1420,237.0901, 223.1475,209.0952	+H,+Na	cryptotanshinone		√
224	25.16	295.1317	$C_{19}H_{18}O_3$	-4.0	—	277.1215,262.0981, 249.1265,234.1030, 206.1083,191.0851	+H	isotanshinone II_A or its isomer		√
225	24.29	299.2012	$C_{20}H_{28}O_2$	-1.6	181.99	283.1724,227.1068, 183.5067	-H	sugiol or its isomer		√
226	24.41	295.2278	$C_{18}H_{32}O_3$	0.2	—	277.2216,227.1087	-H	13-hydroxy-9,11-octadecadienoic acid or its isomer	√	
227	24.92	293.2117	$C_{18}H_{30}O_3$	1.8	180.94	272.9935,244.9802, 227.0000,185.1232	-H	hydroxy-octadecatrienoic acid or its isomer	√	
228	25.64	281.1545	$C_{19}H_{22}O_2$	-0.8	—	265.1230,259.1926, 228.9823	-H	miltirone or its isomer		√
229[a]	25.96	295.1319	$C_{19}H_{18}O_3$	-3.4	165.28/198.38	277.1213,252.0768, 235.0742,221.1315	+H,+Na	tanshinone IIA		√
230	27.38	271.2271	$C_{16}H_{32}O_3$	2.8	178.09	253.2220,225.2214, 197.1883,178.9979	-H	methoxypterocarpane or its isomer	√	

[a] Components identified with the aid of reference compounds comparison.
